# Inheritance of Rootstock Effects in Avocado (*Persea americana* Mill.) cv. Hass

**DOI:** 10.3389/fpls.2020.555071

**Published:** 2020-12-23

**Authors:** Paula H. Reyes-Herrera, Laura Muñoz-Baena, Valeria Velásquez-Zapata, Laura Patiño, Oscar A. Delgado-Paz, Cipriano A. Díaz-Diez, Alejandro A. Navas-Arboleda, Andrés J. Cortés

**Affiliations:** ^1^Corporación Colombiana de Investigación Agropecuaria (AGROSAVIA)—CI Tibaitatá, Mosquera, Colombia; ^2^Department of Microbiology and Immunology, Western University, London, ON, Canada; ^3^Department of Plant Pathology and Microbiology, Interdepartmental Bioinformatics and Computational Biology, Iowa State University, Ames, IA, United States; ^4^Corporación Colombiana de Investigación Agropecuaria (AGROSAVIA)—CI La Selva, Rionegro, Colombia; ^5^Facultad de Ingenierías, Universidad Católica de Oriente—UCO, Rionegro, Antioquia

**Keywords:** heritability, grafting, scion, fruit tree, rootstock-scion interaction, genetic prediction

## Abstract

Grafting is typically utilized to merge adapted seedling rootstocks with highly productive clonal scions. This process implies the interaction of multiple genomes to produce a unique tree phenotype. However, the interconnection of both genotypes obscures individual contributions to phenotypic variation (rootstock-mediated heritability), hampering tree breeding. Therefore, our goal was to quantify the inheritance of seedling rootstock effects on scion traits using avocado (*Persea americana* Mill.) cv. Hass as a model fruit tree. We characterized 240 diverse rootstocks from 8 avocado cv. Hass orchards with similar management in three regions of the province of Antioquia, northwest Andes of Colombia, using 13 microsatellite markers simple sequence repeats (SSRs). Parallel to this, we recorded 20 phenotypic traits (including morphological, biomass/reproductive, and fruit yield and quality traits) in the scions for 3 years (2015–2017). Relatedness among rootstocks was inferred through the genetic markers and inputted in a “genetic prediction” model to calculate narrow-sense heritabilities (*h*^2^) on scion traits. We used three different randomization tests to highlight traits with consistently significant heritability estimates. This strategy allowed us to capture five traits with significant heritability values that ranged from 0.33 to 0.45 and model fits (*r*) that oscillated between 0.58 and 0.73 across orchards. The results showed significance in the rootstock effects for four complex harvest and quality traits (i.e., total number of fruits, number of fruits with exportation quality, and number of fruits discarded because of low weight or thrips damage), whereas the only morphological trait that had a significant heritability value was overall trunk height (an emergent property of the rootstock–scion interaction). These findings suggest the inheritance of rootstock effects, beyond root phenotype, on a surprisingly wide spectrum of scion traits in “Hass” avocado. They also reinforce the utility of polymorphic SSRs for relatedness reconstruction and genetic prediction of complex traits. This research is, up to date, the most cohesive evidence of narrow-sense inheritance of rootstock effects in a tropical fruit tree crop. Ultimately, our work highlights the importance of considering the rootstock–scion interaction to broaden the genetic basis of fruit tree breeding programs while enhancing our understanding of the consequences of grafting.

## Introduction

How different genomes interact to shape a unique phenotype has been one of the most pervasive questions in quantitative genetics and molecular evolution (Lynch, 2007; Bijma, 2013; Fisher and Mcadam, 2019). Horizontal gene transfer (Bennetzen, 1996) and allopolyploidy (Abbott et al., 2013) are often regarded as the typical processes that lead to the interaction of various genomes within a single organism. However, a commonly disregarded yet ancient process that also produces genetic chimeras is grafting, which refers to the agricultural practice that joins the root system (rootstock) of one plant, usually a woody crop, to the shoot (scion) of another (Warschefsky et al., 2016; Gautier et al., 2019; Bartusch and Melnyk, 2020). Grafting began with the earliest tree crops (i.e., olive, grape, and fig) and quickly expanded to several Rosaceae (i.e., apple, plum, pear, and cherry). Modern grafting is crucial for the clonal propagation of fruit trees (e.g., avocado, citrus, grapevine and peach) and the establishment of seed orchards for the wood industry—i.e., pines, teak (Tuskan et al., 2018). Grafting is common in a phylogenetically diverse assortment of fruit and forest tree species, so it offers an irreplaceable experimental playground to study the rootstock–scion interaction (Albacete et al., 2015).

Grafting is typically utilized to merge resilient rootstocks to clonal scions that produce the harvested product, either fruits or wood. Grafting side steps the bottlenecks of breeding woody perennials (Badenes et al., 2016), primarily associated with their outcrossing reproductive system and prolonged juvenile phases (Warschefsky et al., 2016). The root phenotype may confer direct resilience to root pest and pathogens (Cháves-Gómez et al., 2020) as well as to abiotic stresses (He et al., 2020; Martínez-García et al., 2020) such as drought, flooding, and salt soil conditions (Gautier et al., 2019). Rootstocks can also induce less trivial scion morphological changes such as dwarfing and precocity, and even alter yield traits (Egea et al., 2004; Picolotto et al., 2010; Madam et al., 2011; Expósito et al., 2020; Kviklys and Samuolienė, 2020). Rootstock effects can go further and influence properties typically attributed to the clonal scion such as fruit sensorial and nutritional quality—e.g., texture, sugar content, acidity, pH, flavor, and color (Giorgi et al., 2005; Gullo et al., 2014; Balducci et al., 2019), cold tolerance and shoot pest and pathogen resistance (Rubio et al., 2005; Goldschmidt, 2014). These combined effects are influenced by phylogenetic distance and stem anatomy (Wulf et al., 2020) and are mechanistically due to large-scale movement of water, proteins, and nutrients (Little et al., 2016) or long-distance signaling (Lu et al., 2020) via hormones, messenger RNAs, and small RNAs (Wang et al., 2017; Loupit and Cookson, 2020; Rasool et al., 2020). Despite shared physiological processes account for the overall trait variation, the interconnection of all contributing variables (i.e., rootstock genotype, scion genotype, and environment) obscures individual contributions to phenotypic variation (Albacete et al., 2015; Warschefsky et al., 2016). Therefore, an explicit estimation of rootstock effects (i.e., rootstock-mediated heritability) would be a major advance to speed-up tree breeding programs and discern the consequences of grafting.

Narrow-sense heritability (*h*^2^), or the proportion of phenotypic variance among individuals in a population due to genetic effects, is regarded as a baseline of any breeding program (Holland et al., 2003) because it ensures that additive genetic gains are maximized per unit time by optimizing breeding and selection cycles (Dieters et al., 1995). However, heritability estimation in grafted trees has been hampered by their perennial nature and the complexity of the rootstock–scion interaction. A modern pedigree-free marker-based approach to estimate heritability on populations of mixed ancestry (Frentiu et al., 2008; Wilson et al., 2010) is the so-called “genetic prediction” model (Meuwissen et al., 2001; Crossa et al., 2017). This approach relies on genetic-estimated relatedness (Lynch and Ritland, 1999) and mixed linear predictors (i.e. genomic best linear unbiased prediction, gBLUP) to estimate the additive genetic contribution to phenotypic trait variation and thus trait heritability (Milner et al., 2000; Kruuk, 2004; Berenos et al., 2014). Here, we expanded this model to a grafted clonal fruit crop by phenotyping traits at the tree level and genotyping seedling rootstocks to trace back common origins from local “plus tree” donors.

An important fruit tree crop that is nowadays seeing an unprecedented expansion (Clavijo and Holguín, 2020) in tropical and subtropical areas is avocado (*Persea americana* Mill.) cv. Hass. Avocado originated in Central America from where it expanded southward to the northwest Andes, leading to three horticultural races, mid-altitude highland Mexican (*P. americana* var. *drymifolia* Schlecht. et Cham. Blake) and Guatemalan (*P. american*a var. *guatemalensis* L. Wms.) races, and lowland West Indian (*P. americana* var. *americana* Mill.) race (Bergh and Ellstrand, 1986). Previous research about the effect of selected avocado rootstock over crop performance has shown that trees of the same variety grafted to Mexican or Guatemalan race rootstocks differ in their susceptibility to *Phytophtora cinnamomi* (Smith et al., 2011; Reeksting et al., 2016; Sánchez-González et al., 2019), in their mineral nutrient uptake (Bard and Wolstenholme, 1997; Calderón-Vázquez et al., 2013) and in their response to salinity (Mickelbart and Arpaia, 2002; Raga et al., 2014). For instance, Bernstein et al. (2001) demonstrated that even among selected rootstocks chosen by exhibiting excellent fruit production under elevated NaCl condition, there is a wide range of growth sensitivities that results in growth inhibition or growth stimulation under salt levels typically found at commercial fields. Furthermore, rootstocks from different races change the carbohydrate accumulation profile in trees of the same variety, which is known to drive productivity (Whiley and Wolstenholme, 1990) and can ultimately influence alternate bearing, yield components, and nutrition on “Hass” (Mickelbart et al., 2007). Rootstocks can even affect postharvest anthracnose development (Willingham et al., 2001) and the blend of biogenic volatile organic compounds emitted by “Hass” (Ceballos and Rioja, 2019), which could be associated with scion pest attraction. Besides, because rootstock–scion interaction works both ways, different scions may also have distinct effects on avocado rootstock traits, such as arbuscular mycorrhizal, root hair development (Shu et al., 2017), and plant–soil exchanges (Sedlacek et al., 2014).

Despite that several studies have provided evidence of avocado rootstock effects on “Hass” crop performance, the genetic identity and the adaptive potential of the rootstocks that are already planted or are being offered by the nurseries remain a major knowledge gap due to their admixed origin. Additionally, because many “Hass” avocado orchards are yet to be established worldwide in upcoming years, demand for selected rootstocks is reaching its peak, but explicit rootstock effect estimates are still lacking. Hence, our objective was to quantify the inheritance of rootstock effects on “Hass” avocado traits by expanding a “genetic prediction” model to open-pollinated (OP) non-Hass seedling rootstocks from various provenances. This will enlighten the consequences of grafting while enhancing avocado rootstock breeding.

## Materials and Methods

### Plant Material and Orchards Management

**FIGURE 1:**
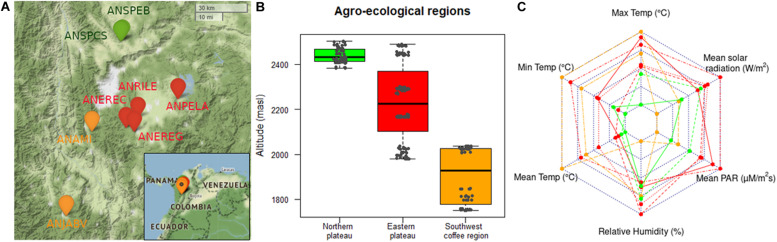
Orchards of “Hass” avocado sampled as part of this study in the northwest Andes of Colombia (province of Antioquia). A total of eight orchards with comparable management for the exportation market spanned three agroecological regions, two in the dairy Northern Andean highland plateau (in green), four in the Eastern Andean highland plateau (in red), and two in the Southwest coffee region (in orange). Thirty trees distributed in six blocks were chosen at each orchard, for a total of 240 trees grafted on OP non-Hass seedling rootstocks (Supplementary Table 1). Orchards names are depicted in **(A)**, whereas altitudinal profile per agroecological region is shown in **(B)**, and key environmental descriptors per orchard (lines) and region (colors) are potted in **(C)**. Map was done in R v.3.4.4 (R Core Team) using *leaflet* package. *Temp* and *PAR* in **(C)** respectively stand for temperature and photosynthetically active radiation.

Avocado cv. Hass production areas in Colombia are widely variable in terms of environmental factors such as altitude, solar radiation, relative humidity, temperature, and precipitation. This variability affects avocado production in terms of agronomic behavior, productivity, yield, and fruit quality. To discern rootstock-mediated heritability from environmental drivers, we chose eight commercial orchards of avocado cv. Hass at the Antioquia province that have been in production for the exportation market for 5 years since 2016. Orchards had comparable nutrient management (Tamayo-Vélez et al., 2018), allowing for litter decomposition (Tamayo-Vélez and Osorio, 2018) without irrigation or hormone supplements, and were only subjected to annual light correctional pruning. Orchards spanned three different agroecological regions, two in the dairy Northern Andean highland plateau, four in the Eastern Andean highland plateau, and two in the Southwest coffee region (Figure 1). At each orchard, we selected six randomly distributed blocks with five trees per block (average spacing 7 × 6 m), for a total of 240 trees grafted on OP non-Hass seedling rootstocks (Supplementary Table 1). Sites and climate were mapped in R v.3.4.4 (R Core Team) using the *leaflet* and *fmsb* packages.

### Measurements of Phenotypic Traits

All 240 trees grafted on seedling rootstocks were measured in 2016 for eight morphological traits. Tree and trunk height were recorded, as well as the height of the rootstock and the scion, using the grafting scar as reference. Rootstock and the scion perimeter were measured below and above the grafting scar, too. Trunk perimeter at the grafting scar and a quantitative score following Webber (1948) were visual proxies for the anatomical quality of the grafting. Furthermore, three biomass/reproductive traits were measured weekly from 2015 to 2017. Flowers and fruits were marked in four cardinally oriented branches, whereas fallen leaves, flowers, and fruits were collected from nets placed aboveground and weighted to estimate the total number of leaves, flowers, and fruits according to Salazar-García et al. (2013). Complete biomass/reproductive measures were possible for 144 trees across all 3 years.

Meanwhile, the annual harvest from 2015 to 2017 was cataloged in nine categories according to fruit quality. The number of fruits with exportation quality was recorded as a combined trait for yield and quality. If a fruit did not reach quality for exportation, the reason why it was discarded was also annotated. In this sense, the number of fruits that exhibited mechanical or sun damage was recorded as well as fruits with signs of damage by pests such as scarab beetles—*Astaena pygidialis* (Holguín and Neita, 2019), thrips (*Frankliniella gardeniae*), or *Monalonion* spp. Furthermore, fruits may not be suitable for exportation due to other imperfections such as low weight, early ripening, or stalk-cut below pedicel, which were annotated, too. Complete harvest categories were possible for 161 trees across all 3 years. Trait differences among trees at distinct agroecological regions and orchards were determined via the Wilcoxon rank-sum test for each trait. Additionally, Pearson correlations among phenotypic traits and between them and altitude were calculated using the *PerformanceAnalytics* package. All analyzes were carried out in R v.3.4.4 (R Core Team).

### Genetic Screening

Healthy roots from grafted avocado trees were sampled, washed, and stored at −20°C. Total genomic DNA was extracted from roots following Cañas-Gutiérrez et al. (2015). DNA quality was checked on a NanoDrop 2,000 (Thermo Fisher Scientific, United Kingdom). A total of 13 microsatellite markers [simple sequence repeats (SSRs)], originally designed by Sharon et al. (1997) and Ashworth et al. (2004), were chosen for their high polymorphism information content following estimates by Alcaraz and Hormaza (2007) (Supplementary Table 2). Forward primers were labeled with WellRed fluorescent dyes at the 5′ end (Proligo, France). SSR markers were multiplexed in three PCR amplifications ran on a Bio-Rad thermocycler (Bio-Rad Laboratories, Hercules, CA, United States) using the GoTaq^®^ Flexi DNA Polymerase kit (Promega, United States). Reaction volumes and thermocycling profiles were set according to the manufacturer’s instructions. Resulting PCR products were evaluated for thermocycling reaction efficiency on 1.5% agarose gels and then analyzed using capillary electrophoresis in a CEQ 8,000 capillary DNA analysis system (Beckman Coulter, Fullerton, CA, United States) at Corporación para Investigaciones Biológicas (CIB, Colombia). Allele sizes were estimated in base pairs with Peak Scanner (Thermo Fisher Scientific, United States), allowing for a maximum of two alleles per sample. High quality genotype data were possible for 188 trees (Supplementary Table 1), for which DNA extraction, SSR amplification, and allele scoring succeed.

### Population Structure and Relatedness Estimation

The accuracy of heritability estimates is dependent on population stratification and sample relatedness within populations (Berenos et al., 2014; Cortés et al., 2014; Sedlacek et al., 2016). Therefore, we first assessed population structure with an unsupervised Bayesian clustering approach implemented in STRUCTURE software (Pritchard et al., 2000), which determines a *Q* matrix of population admixture across various *K*-values of possible subpopulations found in a sample of genetic diversity more robustly than other clustering methods (Stift et al., 2019). A total of five independent runs were used for each *K* value from *K* = 2 to *K* = 7 using an admixture model and 100,000 Markov chain Monte Carlo replicates with a burn-in of 50,000. Permutations of the output of STRUCTURE were performed with CLUMPP software (Jakobsson and Rosenberg, 2007) using independent runs to obtain a consensus matrix based on 15 simulations. The final structure of the population was determined based on cross-run cluster stability and the likelihood of the graph model from Evanno et al. (2005), and the admixture index (a measure of inter-population outcrossing) was recorded for each sample at the optimum *K* value.

We further explored within-population relatedness using Lynch and Ritland (1999) relatedness estimator because this is the most commonly used, which makes eventual comparisons with other studies easier. Computations were implemented in SPaGeDi v. 1.4 software (Hardy and Vekemans, 2002). Diagonal elements of the matrix were set to one as they describe the relatedness of a genotype with itself. Relatedness estimates between the 17,578 pairwise comparisons were summarized using *hist* and *summary* functions in the R v.3.4.4 (R Core Team) environment.

### Estimation of Genetic Rootstock Effects on Scion Traits

We used a mixed linear model to predict phenotypic scores for each trait from the rootstock genotypic information following de los Campos et al. (2009). Due to the clonality of the scion (i.e., absence of genetic variance), it is feasible to disentangle the effect of rootstock genetics into the scion phenotype. Thus, we used the additive model described in Eq. 1 to predict the phenotypic value based on the rootstock’s genotype.

(1) $$y_{i}=\mu +\sum_{j=1}^{m}x_{i\,j}\,\beta_{j}+e$$

where *y*_*i*_ is the score predicted for each trait for the *i*th individual, μ is the mean of each trait in the entire population, *x*_*ij*_ is the relatedness between the *i*th and the *j*th individuals, following Lynch and Ritland (1999) and Cros et al. (2015), *m* is the total number of samples, β_*j*_ is the estimated effect for the relatedness to the *j*th individual on the trait, and *e* is the estimated error associated with the trait. By using Lynch and Ritland’s (1999) relatedness estimate within Eq. 1, we can enlarge the set of variables to 188. However, we still considered a simpler model using the genetic markers by themselves instead of the relatedness matrix so that *x*_*ij*_ was the genotype of the *i*th individual for the *j*th marker and β_*j*_ was the estimated maker effect. To fit these models to our data, we used semi-parametric genomic regression based on reproducing kernel Hilbert spaces regressions methods (Gianola et al., 2006; de los Campos et al., 2010) implemented in the R package *BGLR* (Perez and de los Campos, 2014). We estimated marker effects and the error associated by running for each trait a Gibbs sampler with 10,000 iterations and an initial burn-in of 5,000.

Narrow sense rootstock-mediated heritability scores (*h*^2^) for all traits were computed following de los Campos et al. (2015) equivalent to genomic heritability (Yang et al., 2017), across and at each agroecological region. Marker-based *h*^2^ was calculated as the proportion of phenotypic variance explained by additive effects $\sigma_{a}^{2}$ and the sum of $\sigma_{a}^{2}$ and the random residual $\sigma_{\delta }^{2}$ (Eq. 2). Random residual contained dominance, epistatic and environmental effects that could not be explained by marker-based additive components in Eq. (1).

(2) $$h^{2}=\frac{\sigma_{a}^{2}}{\sigma_{a}^{2}+\sigma_{\delta }^{2}}$$

In addition, we estimated the model fit for each trait as the Pearson correlation (*r*) between each phenotype and the trait’s genetic estimated (Wray and Goddard, 1994) breeding value (GEBV, β*x*_*i*_), based on the rootstocks’ relatedness, as shown in Eq. (3).

(3) $$r=c\,o\,r\,\left(y_{i},\beta \,x_{i}\right)$$

### Permutation Tests on Phenotype and Genotype to Obtain Significance Scores

We used three different permutation strategies to obtain significance scores to validate whether scion traits were affected by rootstocks’ genotypes. We permuted the three separate inputs: (1) the observed phenotypic vector—*y*_*i*_ as in Eq. 1, (2) the matrix of molecular markers genotyped in the rootstocks—*x*_*ij*_ or the genotype of the *i*th individual for the *j*th marker as in Eq. 1, and (3) the matrix of genetic relatedness among rootstocks—*x*_*ij*_ or the relatedness between the *i*th and the *j*th individuals as in Eq. 1. In all cases, we used 50 random permutations without replacement so that the resampling would approximate a random sample (“null” distribution) from the original population. All labels were exchangeable under the null hypothesis. We obtained one-sided *p*-values (type *I* error) for each permutation type, expressed as the proportion of sampled permutations where resultant heritability was larger than the observed heritability estimate. We used this strategy to highlight traits significantly linked with the rootstocks’ genotypes, that is, those for which significant *p*-values (*p* < 0.05) were obtained simultaneously for all three types of permutations.

Finally, to explore whether admixed rootstocks may boost trait variation due to heterotic effects (Isabel et al., 2020), we regressed GEBV (β*x*_*i*_) of traits significantly linked with the rootstocks’ genotypes against the admixture index recorded for each sample at the optimum *K*-value from STRUCTURE computation. Regressions controlled for the agroecological region as random effect via mixed linear models (MLMs) in R’s package *nlme* (Pinheiro et al., 2011).

## Results

### Phenotypic Differences Among Agroecological Regions and Orchards

There were significant differences in the distributions of 15 out of 20 phenotypic traits among different agroecological regions in terms of altitude (Figure 1B), temperature, and radiation (Figure 1C), according to Wilcoxon rank-sum test (*p* < 0.05, Supplementary Table 3). In general, traits recorded at trees in the Southwest coffee region had a distribution shifted to the right compared to trees in the Northern and Eastern Andean highland plateaus. The three measures of trunk perimeter (in the rootstock, scion, and the grafting scar) and the number of fruits with mechanical damage from trees in the Northern plateau had a median higher than trees in the Southwest coffee region and the Eastern plateau. Trees in the Southwest region exhibited higher medians for four out of eight morphological traits (tree height, trunk height, rootstock height, and rootstock compatibility), two out of three biomass/reproductive measures (number of fruits and number of leaves), and five out of nine annual harvest traits (number of fruits with exportation quality, low weight, and sun damage, as well as those damaged by thrips or ripened, *p* < 0.05, Supplementary Table 3).

Meanwhile, there were differences in the distributions of 7, 9, and 19 traits between orchards within the Northern, Southwest, and Eastern agroecological regions, respectively, based on Wilcoxon rank test (*p* < 0.05, Supplementary Table 3). Orchards with the highest trait’s medians were ANSPEB and ANPELA in the Northern and Eastern plateaus, respectively. Details regarding trait distribution differences by regions and orchards are depicted in Supplementary Figures 1–5.

Regarding altitude, there were significant trait differences for 14 out of 20 traits (*p* < 0.05, Supplementary Table 5). For all cases, the correlation with the altitude was negative. The strongest altitudinal correlations were for the rootstock (*r* = −0.61, *p* < 0.05) and trunk (*r* = −0.58, *p* < 0.05) heights and the number of fruits with low weight (*r* = −0.59, *p* < 0.05).

Finally, most of these traits were also significantly correlated with each other. In the group of morphological traits, the highest correlations were between (1) tree height and scion length (*r* = 0.94, *p* < 0.05, Supplementary Figure 6) and (2) the perimeters of the rootstock, scion, and the overall trunk (*r* = 0.8—0.84, *p* < 0.05, Supplementary Figure 6). The three biomass/reproductive traits had medium correlations (*r* = 0.37—0.40, *p* < 0.05, Supplementary Figure 7). For harvest traits, the highest correlations were between (1) the number of fruits with the stalk cut below the pedicel and with damage caused by thrips (*r* = 0.64, *p* < 0.05, Supplementary Figure 8) and (2) the number of fruits with low weight and with exportation quality (*r* = 0.61, *p* < 0.05, Supplementary Figure 8).

### Relatedness and Population Structure Estimates

**FIGURE 2:**
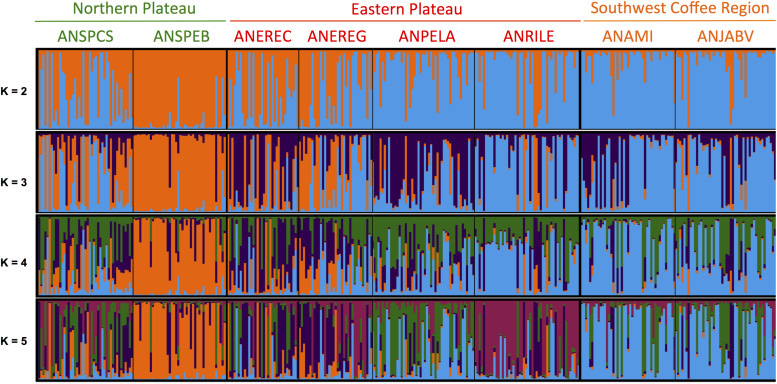
Population structure of seedling rootstocks across eight orchards of “Hass” avocado as inferred with an unsupervised Bayesian clustering approach implemented in STRUCTURE (Pritchard et al., 2000) using 13 SSR markers. A graphical representation of the *Q*-matrix is shown, where each stacked bar corresponds to a seedling rootstock, and within bar colors represent subpopulation assignment probabilities given *K* possible clusters. Orchards are sorted according to the agroecological region, and their names are shown and colored accordingly at the top of the bar plot (following Figure 1). *K*-values of possible subpopulations ranged from 2 to 5. Optimum *K*-value of 3 was determined based on cross-run cluster stability of five independent runs and likelihood of the graph model (Supplementary Figure 9) from Evanno et al. (2005). Higher *K*-values did not contribute further divergence yet increased overall admixture levels. *Q* matrix of population admixture at *K* = 3, and the admixture levels are summarized in Supplementary Table 6. SSR markers (2) were designed by Sharon et al. (1997) and Ashworth et al. (2004) and were prioritized according to their polymorphism information content following Alcaraz and Hormaza (2007).

Evaluation of population structure using an unsupervised Bayesian clustering approach implemented in STRUCTURE with *K* = 2 to *K* = 10 subpopulations resulted in an ideal *K*-value of three subpopulations (Supplementary Figure 9) based on the increases in likelihood ratios between runs using Evanno’s delta *K* statistic (Evanno et al., 2005) and cross-run cluster stability. Points of inflection were not observed for the log-likelihood curve, but a smaller increase of the likelihood was found when comparing *K* = 3 and *K* = 4 to other *K*-values. However, cross-run cluster stability did not result in the split of a fourth subpopulation compared with *K* = 3. The separation of the subpopulations at each *K*-value is informative and, therefore, is presented in Figure 2. At the first level of subpopulation separation, *K* = 2, one orchard from the Northern plateau (ANSPEB) split, whereas the other orchard from the Northern plateau (ANSPCS) and two from the Eastern plateau (ANEREC and ANEREG) revealed high levels of admixture. At *K* = 3, two orchards from the Eastern plateau (ANEREC and ANPELA) differentiated from the others by high levels of admixture. At *K* = 4, all subpopulations were admixed for the fourth subpopulation except ANSPEB, which differentiated homogeneously since *K* = 2. Higher *K*-values did not contribute further divergence but increased overall admixture levels.

**FIGURE 3:**
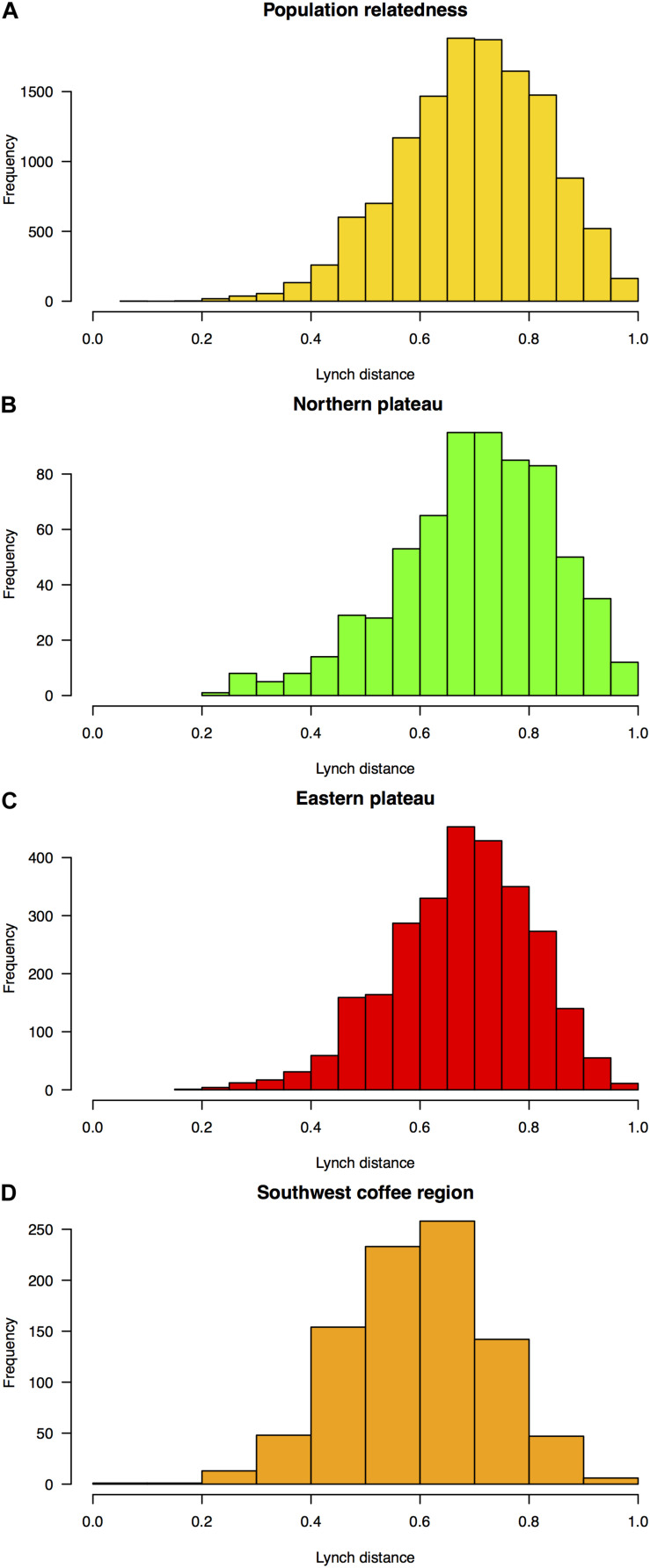
Frequency distribution of pairwise marker-based estimates of Lynch and Ritland (1999)’s relatedness among OP non-Hass seedling rootstocks from eight “Hass” avocado orchards. Relatedness distributions are shown among **(A)** all rootstocks (yellow), and rootstocks in the **(B)** dairy Northern Andean highland plateau (green), **(C)** Eastern Andean highland plateau (red), and **(D)** Southwest coffee region (orange). Relatedness estimates were inputted in a “genetic prediction” additive mixed linear model according to de los Campos et al. (2009) to compute pedigree-free (Frentiu et al., 2008; Wilson et al., 2010) rootstock-mediated narrow-sense heritability (*h*^2^) for 20 traits (Table 1 and Figure 4).

Admixture levels at *K* = 3 in the orchard of the Northern plateau (ANSPCS) and the two orchards of the Eastern plateau (ANEREC, ANEREG) that exhibited high heterogeneity from *K* = 2 were significantly higher than in the rest (0.26 ± 0.05 vs. 0.18 ± 0.03, *p* < 0.05, Supplementary Table 6). The more dissimilar orchard (ANSPEB) was the less admixed (0.10 ± 0.03). Overall genetic relatedness, according to Lynch and Ritland (1999), ranged from 0.2 to 1.0, spanning a wide spectrum of relatedness values comparable across all three agroecological regions (Figure 3). Therefore, mixed ancestry and various levels of family stratification (Barton et al., 2019) fulfill prerequisites for heritability estimates.

### Genetic Heritability and Predictive Ability

**FIGURE 4:**
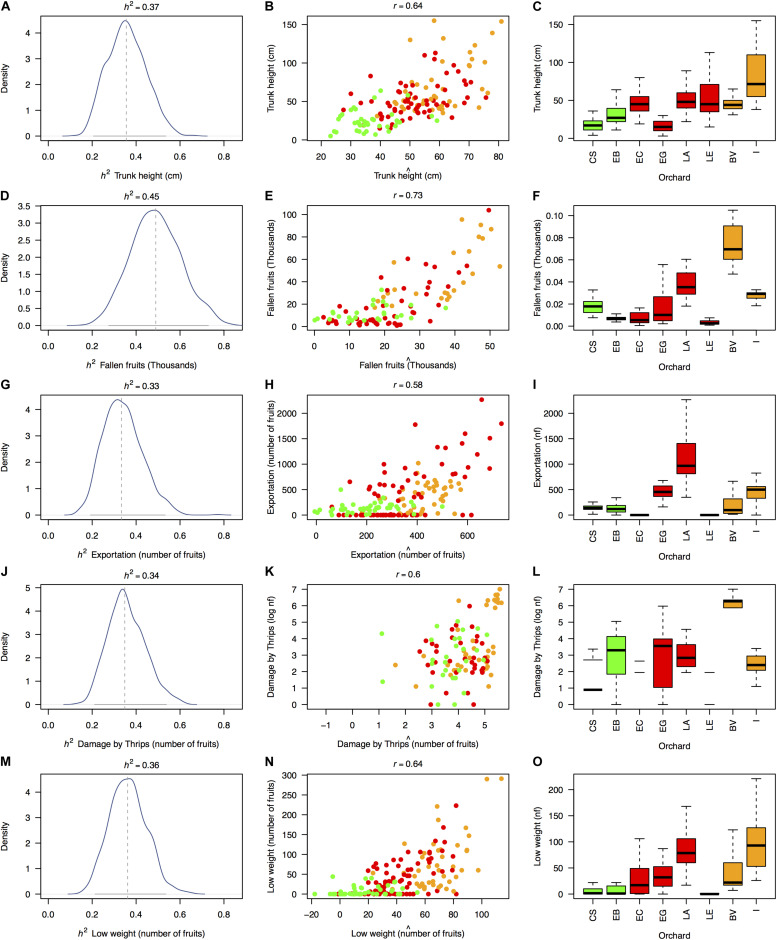
Significant estimates of narrow-sense rootstock-mediated heritability (*h*^2^) in 5 of the 20 measured traits based on a “genetic prediction” model calibrated with Lynch and Ritland (1999)’s relatedness matrix among OP non-Hass seedling rootstocks from eight “Hass” avocado orchards. Depicted traits (rows, **A–O**) are those for which significant *p*-values (*p* < 0.05) were simultaneously obtained for three different permutation strategies (of the phenotypic vector, the matrix of molecular markers and the matrix of genetic relatedness among rootstocks, Table 1), although the graphical results only reflect estimates obtained after permuting the relatedness matrix. First column of figure panels **(A,D,G,J,M)** shows the posterior distribution for the rootstock-mediated heritability (*h*^2^) estimates as well as their mean (dashed vertical gray line) and 95% confidence interval (continuous horizontal gray line). Second column of figure panels **(B,E,H,K,N)** reflects the model fits (*r*) expressed as the correlation between the observed trait phenotype (*y*_*i*_) and the model’s estimated breeding value (β*x*_*i*_) (Eq. 3). Third column of figure panels **(C,F,I,L,O)** recalls the trait distribution across orchards (from Supplementary Figures 1–5). Dots and boxplots are colored according to Figure 1, as follows: dairy Northern Andean highland plateau in green, Eastern Andean highland plateau in red, and Southwest coffee region in orange. Estimates of *h*^2^ and *r* are derived from an additive mixed linear model according to de los Campos et al. (2009). *nf* stands for number of fruits.

**FIGURE 5:**
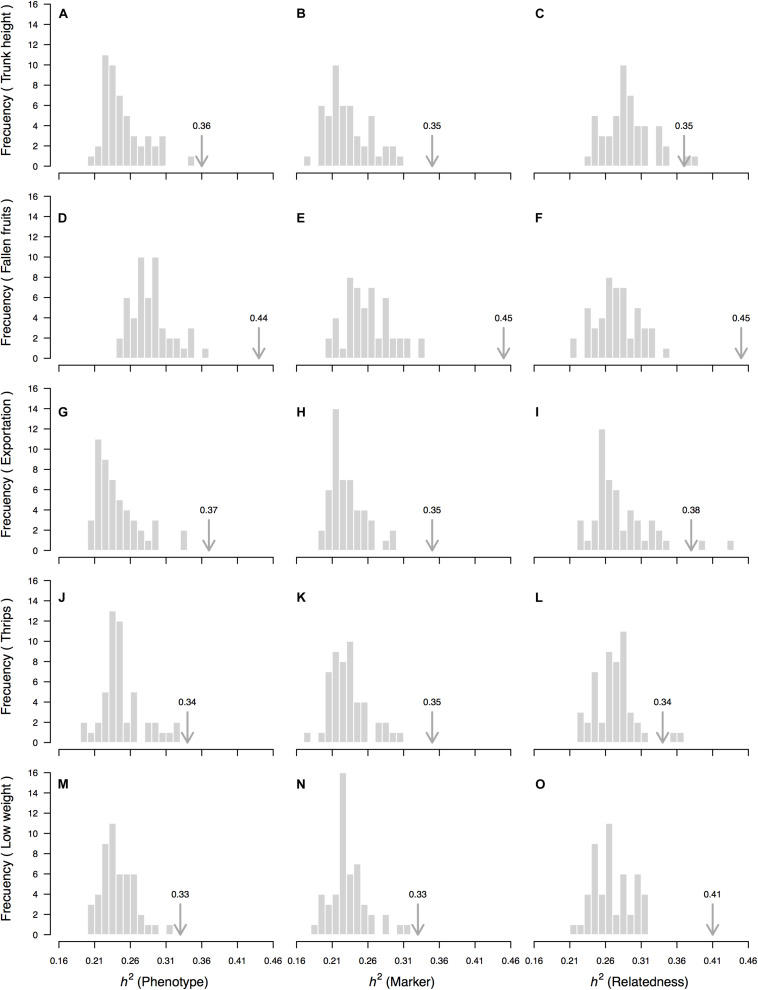
“Null” distributions (random sample) of the rootstock-mediated heritability (*h*^2^) estimate for 5 of the 20 measured traits based on a “genetic prediction” model calibrated with Lynch and Ritland (1999)’s relatedness matrix among OP non-Hass seedling rootstocks from eight “Hass” avocado orchards. Depicted traits (rows, **A–O**) are those for which significant *p*-values (*p* < 0.05) were simultaneously obtained for three different permutation strategies—of the phenotypic vector (first column of figure panels, **A,D,G,J,M**), the matrix of molecular markers (second column of figure panels, **B,E,H,K,N**) and the matrix of genetic relatedness among rootstocks (third column of figure panels, **C,F,I,L,O**). In all cases, 50 random permutations without replacement were used. Average rootstock-mediated heritability (*h*^2^) estimates (from Table 1) are marked with an arrow. Proportion of sampled permutations where resultant heritability was larger than the observed heritability estimate corresponds to the one-sided *p*-value reported in Table 1.

Estimates of rootstock-mediated heritabilities (*h*^2^) were significant for 5 of the 20 measured traits (Figure 4), regardless of the permutation strategy (Figure 5) and ranged from 0.33 to 0.45 averaged *h*^2^ values with average model fits (*r*) ranging from 0.58 to 0.73 (Table 1). The majority of traits with significant rootstock-mediated heritability were annual harvest traits (number of fruits with exportation quality, low weight, and damages by thrips with average *h*^2^ values of 0.33, 0.36, and 0.34 and average *r* values of 0.58, 0.64, and 0.6, respectively). Only one morphological trait had significant results according to the permutation tests—trunk height with average *h*^2^ and *r* values of 0.37 and 0.64. The number of fruits was the only biomass/reproductive trait with significant results with average *h*^2^ and *r* values of 0.45 and 0.73.

**TABLE 1:** Narrow-sense rootstock-mediated heritability (*h*^2^) estimates for the 20 measured traits from eight “Hass” avocado orchards.

	**Phenotypic traits**	**Phenotypic vector randomization**			**SSR matrix randomization**			**Relatedness matrix randomization**		
		***h*^2^**	***p*-value**	***r***	***h*^2^**	***p*-value**	***r***	***h*^2^**	***p*-value**	***r***
**Morphological traits (2016)**	Tree height (cm)	0.25	0.30	0.48	0.25	0.14	0.48	0.26	0.44	0.49
	Trunk height (cm)	**0.36**	**< 0.01**	**0.64**	**0.35**	**< 0.01**	**0.64**	**0.37**	**0.04**	**0.64**
	Rootstock height (cm)	0.26	0.44	0.52	0.27	0.26	0.52	0.27	0.64	0.52
	Scion height (cm)	0.26	0.14	0.49	0.25	0.06	0.49	0.26	0.52	0.49
	Rootstock perimeter (cm)	0.28	0.28	0.52	0.28	0.06	0.52	0.27	0.68	0.52
	Scion perimeter (cm)	0.26	0.34	0.49	0.26	0.20	0.49	0.26	0.68	0.50
	Trunk perimeter at the grafting scar (cm)	0.28	0.18	0.54	0.29	0.02	0.54	0.29	0.48	0.54
	Rootstock compatibility (Webber, 1948)	0.25	0.70	0.47	0.25	0.38	0.48	0.25	0.92	0.47
**Biomass/reproductive traits (average 2015–2017)**	Number of leaves	0.27	0.68	0.50	0.27	0.34	0.50	0.26	0.88	0.50
	Number of flowers	0.29	0.38	0.53	0.28	0.18	0.53	0.27	0.62	0.53
	Number of fruits (NF)	**0.44**	**< 0.01**	**0.73**	**0.45**	**0.00**	**0.74**	**0.45**	**0.02**	**0.73**
**Harvest traits (average 2015–2017)**	NF with exportation quality	**0.37**	**< 0.01**	**0.65**	**0.33**	**< 0.01**	**0.58**	**0.38**	**0.04**	**0.58**
	NF with mechanical damage	0.28	0.60	0.54	0.23	0.38	0.43	0.23	0.80	0.43
	NF with sun damage	0.28	0.80	0.51	0.24	0.36	0.44	0.24	0.76	0.45
	NF with damage caused by scarab beetles	0.29	0.18	0.56	0.26	0.22	0.50	0.27	0.52	0.50
	NF with damage caused by thrips	**0.34**	**0.02**	**0.62**	**0.35**	**< 0.01**	**0.60**	**0.34**	**0.02**	**0.60**
	NF with damage caused by *Monalonion*	0.34	0.30	0.65	0.27	0.06	0.52	0.27	0.34	0.52
	NF discarded because of low weight	**0.33**	**< 0.01**	**0.62**	**0.35**	**< 0.01**	**0.64**	**0.41**	**0.06**	**0.64**
	NF with early ripening	0.37	0.06	0.66	0.34	0.02	0.59	0.33	0.10	0.59
	NF with the stalk cut below the pedicel	0.45	0.20	0.74	0.26	0.06	0.49	0.26	0.50	0.49

*Heritability (h*^2^*) and model fits (r) estimates were gathered using Lynch and Ritland (1999)’s relatedness matrix inputted in a “genetic prediction” additive mixed linear model, according to de los Campos et al. (2009). One-sided p-values of the observed heritability were estimated using independent permutations of the phenotypic vector, the matrix of molecular markers, and the matrix of genetic relatedness among rootstocks (Figure 5). Consistently significant values are in bold (Figure 4).*

**FIGURE 6:**
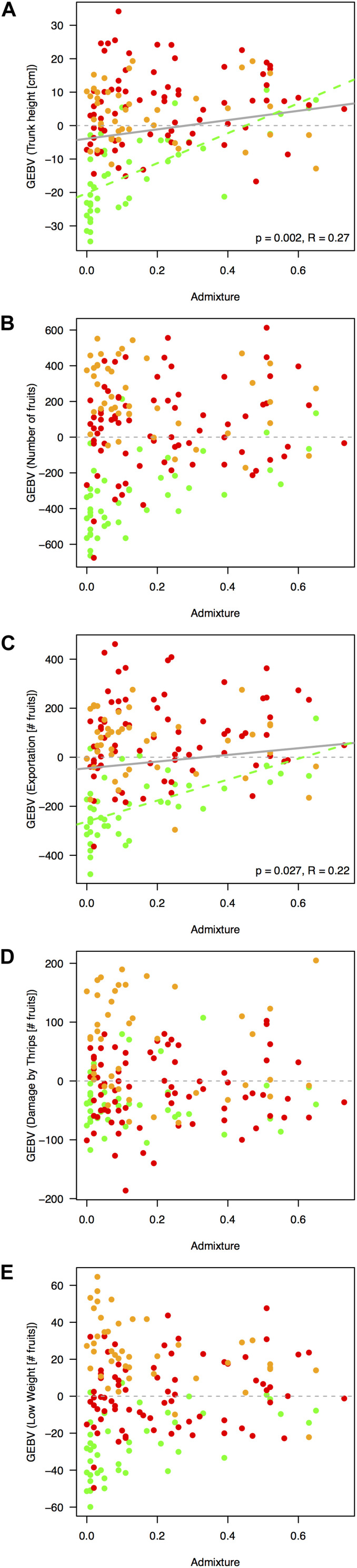
Regressions between rootstock’s breeding (GEBV, β*x*_*i*_) and admixture indices as proxy of heterotic effects. Only traits significantly linked with the rootstocks’ genotypes, after accounting for regional agroecological differences, are depicted, as follows: **(A)** trunk height, **(B)** number of fruits, **(C)** number of fruits with exportation quality, **(D)** number of fruits with damage caused by thrips, and **(E)** number of fruits discarded because of low weight. Overall tendency lines are drawn in gray if significant at a *p*-value threshold of 0.05. Admixture index at an optimum *K*-value of 3 is detailed in Figure 2. Regressions controlled for agroecological region as random effect via MLM models in R’s (v.3.4.4, R Core Team) package *nlme* (Pinheiro et al., 2011). Dots and dashed tendencies are colored according to Figure 1, as follows: dairy Northern Andean highland plateau in green, Eastern Andean highland plateau in red, and Southwest coffee region in orange.

In general, significant morphological and physiological traits had higher *h*^2^ values (*h*^2^ = 0.37 ± 0.01 and *h*^2^ = 0.45 ± 0.01 for trunk height and the number of fruits, respectively) than annual harvest traits (*h*^2^ = 0.33 ± 0.02, *h*^2^ = 0.36 ± 0.01, and *h*^2^ = 0.34 ± 0.01 for the number of fruits with exportation quality, low weight, and damages caused by thrips, respectively). Meanwhile, trait predictability was high, especially for the significant biomass/reproductive trait total number of fruits (*r* = 0.73) and was lowest for the number of fruits with exportation quality (*r* = 0.58). Per-agroecological region *h*^2^ scores were marginally inflated due to decreased environmental variance (Supplementary Figure 10). When considering a model using genetic markers as direct predictors instead of the relatedness matrix, estimates were statistically unpowered for all traits (Supplementary Figure 11). For the five traits significantly linked with the rootstocks’ genotypes (Figures 6A–E), admixed rootstocks marginally enhanced trunk height (*R*^2^ = 0.27, *p* = 0.002, Figure 6A) and number of fruits for exportation (*R*^2^ = 0.22, *p* = 0.027, Figure 6C) after accounting for the agroecological region as a random effect within an MLM framework.

## Discussion

We quantified the genetic effects of avocado seedling rootstocks on 20 “Hass” scion traits using a “genetic prediction” model that related traits’ variation with the SSR identity of rootstocks from eight different orchards. Trees exhibited high levels of admixture across orchards, consistent with rampant gene flow among putative races. Genetic estimates of rootstock-mediated heritability (*h*^2^) were significant for 5 of the 20 measured traits and ranged from 0.33 to 0.45 *h*^2^ with model fits (*r*) between 0.58 and 0.73 across orchards. The only morphological trait that we found having a significant genetic-estimated heritability value was trunk height, likely an emergent property of the rootstock–scion interaction in orchards of the same age only subjected to comparable annual light correctional pruning. Additionally, there were significant rootstock effects for various harvest and quality traits such as total number of fruits, number of fruits with exportation quality, and number of fruits discarded because of low weight and damage by thrips. These findings suggest the inheritance of rootstock effects on a wide spectrum of “Hass” avocado traits relevant for yield, which will be critical to meet the demands of the growing worldwide market.

### Relatedness and Population Admixture Are Consistent With Rampant Gene Flow Among Three Populations

Examination of population structure using an unsupervised Bayesian clustering approach and within-population relatedness using Lynch and Ritland (1999) relatedness estimator are indicative of three major clusters with high levels of admixture that span a wide spectrum of relatedness, allowing for unbiased relatedness-based heritability predictions. These clusters could potentially match the three horticultural races described for avocado, which are mid-altitude highland Guatemalan (*P. americana* var. *guatemalensis* L. Wms.) and Mexican (*P. americana* var. *drymifolia* Schlecht. et Cham. Blake) races, and lowland West Indian (*P. americana* var. *americana* Mill.) race. Previous genetic analyses and screenings of key botanical descriptors have marginally reinforced this race structure (Cañas-Gutiérrez et al., 2019; Cañas-Gutierrez et al., 2019).

Previous genetic characterizations providing tangential signals of horticultural races have used targeted genes (Chen et al., 2009), chloroplast DNA (Ge et al., 2019), SSR (Alcaraz and Hormaza, 2007; Ferrer-Pereira et al., 2017; Boza et al., 2018; Sánchez-González et al., 2020), and SNP markers (Kuhn et al., 2019b; Rubinstein et al., 2019; Talavera et al., 2019), in some cases using gene-bank accessions, such as from the Venezuelan germplasm bank—Instituto Nacional de Investigaciones Agrícolas–Centro Nacional de Investigaciones Agropecuarias (Ferrer-Pereira et al., 2017), the National Germplasm Repository (United States Department of Agriculture—Agricultural Research Service Subtropical Horticultural Research Station) in Miami (Kuhn et al., 2019a,b), and the Spanish germplasm bank (Talavera et al., 2019). Despite some of these analyses captured all three races (Talavera et al., 2019), others exhibited mixed and inconclusive population structure (Cañas-Gutiérrez et al., 2015, 2019; Cañas-Gutierrez et al., 2019). However, modern genomic tools not only have enlightened race substructure (Rendón-Anaya et al., 2019; Talavera et al., 2019) but also provided evidence for the hybrid origin of commercially important varieties such as Mexican/Guatemalan “Hass” avocado (Rendón-Anaya et al., 2019). Our characterization has further highlighted the admixed origin of seedling rootstocks currently used at commercial orchards in the northwest Andes. Persistent admixture due to rampant gene flow is expected for a species that, as avocado, has been subjected to continent-wide animal and human-mediated migration (Bergh and Ellstrand, 1986; Galindo-Tovar et al., 2007; Larranaga et al., 2020), besides being an obligate outcrossing (via protogynous dichogamy, a sequential non-overlapping hermaphroditism in which female function precedes male function).

Regarding economical traits, the Guatemalan race typically has small seeds and exhibits late fruit maturity, whereas Mexican race shows early fruit maturity and cold tolerance. In contrast, the West Indian race has a large fruit size and low oil content (Bergh and Ellstrand, 1986). However, trait differentiation could not be assessed in this study because genotyping was carried out on seedling rootstocks. To evaluate in more detail rootstocks’ fruit phenotype, stooling or layering would need to be induced from rootstocks (Knight et al., 1927; Webster, 1995), a technique normally used for clonal propagation of the desired rootstock rather than high-scale phenotyping. A so far unexplored yet promising alternative would be to calibrate Genomic Prediction (Crossa et al., 2017; Grattapaglia et al., 2018) and Machine Learning (Gianola et al., 2011; Libbrecht and Noble, 2015; Schrider and Kern, 2018) models using high-throughput genotyping (Cortés et al., 2020b) of phenotyped ungrafted avocado trees spanning all three races, to predict rootstocks’ own unobserved phenotypes. Interpolating these predictions and quantitative genetic parameters across the rich ecological continuum of the northern Andean mountains (Madriñán et al., 2013; Valencia et al., 2020), within a multi-climate (Costa-Neto et al., 2020) “enviromic prediction” paradigm (Resende et al., 2020), will be key to target optimum genotype x environment arrangements for yield (Galeano et al., 2012; Blair et al., 2013) and quality (Wu et al., 2020) components, as well as in the face of abiotic (Cortés et al., 2020a) and biotic (Naidoo et al., 2019) stresses imposed by climate change.

### Significant Rootstock Effects for Various Complex Harvest and Quality Traits

Our results suggest the inheritance of rootstock effects on a surprisingly wide spectrum of “Hass” avocado genetically complex traits, mostly spanning economically relevant attributes such as total number of fruits, number of fruits with exportation quality, number of fruits discarded because of low weight, and number of fruits damaged by thrips. The only morphological trait that we found having a significant heritability value mediated by the rootstock was trunk height. Interestingly, all these traits refer to the ability of the rootstocks to impact the phenotype of the grafted scion (i.e., harvest/quality traits), or the entire tree (i.e., trunk height), but not the root phenotype itself (e.g., rootstock height or perimeter). This speaks for a predominant role of the rootstock–scion interaction rather than independent additive effects of each genotype, which is expected when combined effects are mainly due to transport of water and nutrients and large-scale movement of hormones, proteins, messenger RNAs, and small RNAs (Wang et al., 2017).

Previous research about the effect of rootstocks on avocado crop performance has focused on susceptibility to *P. cinnamomi* (Smith et al., 2011; Reeksting et al., 2016; Sánchez-González et al., 2019), mineral nutrient uptake (Bard and Wolstenholme, 1997; Calderón-Vázquez et al., 2013), and response to salinity (Bernstein et al., 2001; Mickelbart and Arpaia, 2002; Raga et al., 2014). However, harvest/quality traits have not been explicitly considered in previous studies to assess rootstock effects on “Hass” avocado. Some indirect mechanistic evidence suggests that rootstocks from different races may affect post-harvest anthracnose development (Willingham et al., 2001), alter carbohydrate accumulation (Whiley and Wolstenholme, 1990), and determine yield components, alternate bearing, and nutrition (Mickelbart et al., 2007) on “Hass” avocado. However, this study contributes new concrete evidence of heritable rootstock effects on key quantitative harvest and quality traits (i.e., total number of fruits, number of fruits with exportation quality, and number of fruits discarded because of low weight and damage by thrips), essential for developing novel rootstock breeding schemes targeting fruit quality in variable mountain ecosystems (Cortés and Wheeler, 2018).

Overwhelming rootstock effects also encourage broadening the genetic basis of current avocado rootstock breeding programs. Across Mesoamerica and northern South America, avocado trees are still cultivated in traditional orchards, backyard gardens, and as living fences. They are consumed at a regional scale and harbor a strong potential to improve fruit quantity and quality, besides tree adaptation, when used as rootstocks in commercial “Hass” orchards (Galindo-Tovar et al., 2007). However, for this to occur, a better comprehension of the consequences of grafting, more concretely the rootstock–scion interaction across traits and environments, needs to be achieved, just as envisioned here.

Meanwhile, in the absence of selected clonal rootstocks, admixed rootstocks seem to enhance productivity traits, such as the number of fruits with exportation quality. This heterotic pattern may be due to dominance and overdominance effects, both of which can increase yield and adaptability after a single generation of admixture (Schilthuizen et al., 2004; Seehausen, 2004). While dominance results from the masking of deleterious recessive alleles by the augmented heterozygosity resulting from admixture, overdominance refers to the increase in aptitude due to additive and epistatic interactions of alleles maintained by balancing selection that would have rarely coincided within the same genotype without admixture. Disentangling between these processes would require mapping allele effects across different (environmental/genomics) contexts.

In the long term, major improvements can be achieved by replacing seedling’s rootstocks with a diverse panel of elite clonal genotypes (Ingvarsson and Dahlberg, 2018). However, tropical avocado plantations in the northern Andes are still in their infancy and will likely remain so during the next decade despite some ongoing efforts to (i) identify superior rootstock genotypes in the face of highly heterogeneous mountainous microenvironmental conditions (Cortés and Wheeler, 2018) and (ii) standardize their propagation via micro-cloning (Ernst, 1999), and double grafting (Frolich and Platt, 1971). Breeding for elite clonal tree genotypes with conventional phenotypic selection usually incurs in progeny-testing phases and several clonal trials (Resende et al., 2012), which may double the breeding cycle length compared to gradual population improvement through recurrent selection and testing (Neale and Kremer, 2011). While locally adapted superior clonal rootstocks are identified and propagated, nurseries will have to rely on OP non-Hass “plus tree” donors of seedling rootstocks. In this context, our study, by quantifying the rootstock mediated heritability in avocado, configures as a first step towards the advance of the rootstock gene pool in a hotspot of wild (Migicovsky and Myles, 2017; Burgarella et al., 2019) and cultivated biodiversity (Pironon et al., 2020) of avocados and related Lauraceae species (Gentry and Vasquez, 1996). The next step is a better tracing of seedling rootstocks from “plus trees” (and seed orchards, yet to be established) to nurseries.

One possible caveat of our heritability estimates refers to the number of fruits damaged by thrips. Despite it is known that rootstocks may affect the blend of biogenic volatile organic compounds emitted by “Hass” (Ceballos and Rioja, 2019) and therefore influence scion pest attraction, — or repellence (Kainer et al., 2018); in our study, thrips’ pressure was not homogeneous across nor within orchards. In other words, different rootstocks were not equally exposed to the pest, meaning that the phenotypic vector and the relatedness matrix were fortuitously unbalanced within the “genetic prediction” model. This trend was not observed for any of the other significantly rootstock-inherited traits. Therefore, to validate the rootstock-mediated genetic-estimated heritability values obtained for the number of fruits damaged by thrips, an oncoming controlled experiment would require capturing volatiles across grafted “Hass” trees, all exposed to constant pressure by thrips.

### Relatedness Reconstruction With SSR Markers Allows for Genomic-Type Predictions

SSRs may not be sufficient to describe a polygenic basis, but they can capture a wide spectrum of samples’ relatedness. Heterogeneity in the samples’ relatedness is essential to calibrate a “genetic prediction” model when highly related or unrelated samples are not sufficiently contrasting by themselves. The molecular relationship matrix that we estimated following Lynch and Ritland (1999) and Cros et al. (2019) was adequately heterogeneous. In this way, our genetic prediction managed to include both family effects and Mendelian sampling terms while simultaneously expanding the number of variables from 13 up to 188, increasing the predictive model accuracy (Zhang et al., 2019).

SSRs’ high mutation rate (Ellegren, 2004) and polymorphism content (Cortés et al., 2011; Blair et al., 2012) allow utilizing this type of marker to trace the nature of the rootstock gene pool and disclose the relatedness matrix. This way, it becomes feasible to compute the additive genetic variance of quantitative traits under a “genetic prediction” model (Cros et al., 2015, 2019) without *a priori* knowledge of the parental and family ancestry. Replacing an unknown pedigree by marker-inferred pairwise relatedness between individuals (Lynch and Ritland, 1999) makes viable pedigree-free heritability estimation (Frentiu et al., 2008; Wilson et al., 2010), a major accomplishment in perennials. This strategy recurs to variation across distinct genetically estimated kinship levels (Milner et al., 2000; Kruuk, 2004; Berenos et al., 2014) and not just within and between families (Falconer and Mackay, 1996; Walsh, 2008). However, caution must be taken when extending this approach to other systems for which family heterogeneity is insufficient. Luckily in our case, the relatedness matrix was adequately variable, embracing various families and degrees of relationship, partly due to the fact that nurseries mix seedling rootstocks from OP non-Hass “plus tree” donors of various provenances.

Despite SSRs’ utility, these markers will be limited when trying to assess the genomic architecture of complex traits (Hirschhorn and Daly, 2005) or when calibrating marker-based infinitesimal Genomic Selection models (Kumar et al., 2012, 2015, 2019; Muranty et al., 2015; Crossa et al., 2017). To reveal the rootstock-mediated genomic architecture of key traits, genome-wide association (Khan and Korban, 2012) has to assume some allelic variants are in linkage disequilibrium (LD) (Blair et al., 2018) with causal variants (Hirschhorn and Daly, 2005; Morris and Borevitz, 2011; Tam et al., 2019) that impact scion’s phenotype (Minamikawa et al., 2017). Likewise, predictive rootstock breeding needs to assume that quantitative traits are regulated by infinitive low-effect additive causal variants in LD with many genetic markers (Crossa et al., 2017). Infrequent SSR markers, despite highly polymorphic, are unlikely to be found in LD with any of these variant types (Slatkin, 2008). So, abundant and easily scored SNP markers (Kelleher et al., 2012) will be needed for a deeper comprehension of the rootstock–scion interaction and to enhance its factual utilization for breeding purposes.

### Next Steps to Deepen Our Understanding of the Rootstock–Scion Interaction

To expand our knowledge on the extent of the rootstock–scion interaction and speed up fruit tree breeding programs (Kumar et al., 2020; Peng et al., 2020; Santantonio and Robbins, 2020), further heritability estimates should be gathered on contrasting traits using multi-environment (Crossa et al., 2019; Costa-Neto et al., 2020) provenance (“common garden”) and progeny trials with diverse panels of seedling and clonal rootstocks. The “genetic prediction” model used here to estimate pedigree-free heritabilities (Milner et al., 2000; Kruuk, 2004; Frentiu et al., 2008; Wilson et al., 2010; Berenos et al., 2014), or alternatively indirect genetic effect (IGE) models (Bijma, 2010, 2013; Fisher and Mcadam, 2019), may be extended to field trials at a low genotyping cost, as few polymorphic SSR markers are enough to span the genetic relatedness gradient. This model suggested evidence that rootstock’s influences transcend the root phenotype and can directly impact the phenotype of the grafted scion for economically important traits. Therefore, widening the spectrum of traits under screening for rootstock-mediated heritability will be essential to optimize rootstock selection and the overall genetic value of nurseries’ grafted material in the genomic era (Khan and Korban, 2012; Meneses and Orellana, 2013; Iwata et al., 2016).

On the other hand, rootstock–scion interaction also implies that different scions may have distinct effects on rootstock traits, such as arbuscular mycorrhizal and root hair development (Shu et al., 2017). Studying this type of interaction would require factorial designs in which different clonal scions are grafted ideally on clonally propagated rootstocks—e.g., via double grafting (Frolich and Platt, 1971) or micro-cloning (Ernst, 1999), or alternatively on half-sib families of seedling rootstocks. This way, new scion effects can be revealed while optimizing the rootstock–scion combination. Meanwhile, a new generation of multi-year “genetic prediction” (Crossa et al., 2019; Roudbar et al., 2020) and IGE models – as carry out in social contexts (Feldman et al., 2017; Santostefano et al., 2017; Han et al., 2018), may expand our understanding of how plants graft while pivoting fruit tree breeding programs. We look forward to seeing similar approaches applied on other woody perennial fruit crops and orphan tropical and subtropical native trees.

Besides quantifying rootstock and scion effects using quantitative genetic approaches, a more mechanistic understanding of the consequences of grafting is desirable by applying tools from the “omics” era (Barazani et al., 2014; Wang et al., 2017; Guillaumie et al., 2020). Genotyping-by-sequencing (Elshire et al., 2011; Cortés and Blair, 2018), re-sequencing (Fuentes-Pardo and Ruzzante, 2017), RNAseq (Jensen et al., 2012; Sun, 2012; Reeksting et al., 2016) and single-cell sequencing (Tang et al., 2019) across different tissues of the grafted tree, including the graft interface (Cookson et al., 2019), will enable understanding the genetic architecture of rootstock-mediated traits and the rootstock–scion interaction. Ultimately, these approaches may help discern among additive and combined processes how plant tissues and physiological (Loupit and Cookson, 2020; Rasool et al., 2020) processes (such as water and nutrients uptake and transport, hormone production and transport, and large-scale movement of molecules) behave during grafting.

## Conclusion

Grafting typically enables side-stepping the bottlenecks of breeding woody perennials, mainly concerning their prolonged juvenile phases and outcrossing reproductive systems. Avocado cv. Hass plantations are currently experiencing rampant growth in tropical and subtropical areas, where grafting heavily relies on non-Hass OP seedling rootstocks. However, the individual contribution of the rootstock–scion interaction to phenotypic variation still hampers avocado rootstock breeding and prevent unveiling the consequences of grafting. Throughout this study, we screened 240 grafted trees for 20 phenotypic traits and 13 SSR markers in the seedling rootstocks. This way, we identified five traits with genetic-estimated rootstock-mediated narrow-sense heritability scores significantly different from zero, given three stringent permutation strategies. Because four of these traits were related to fruit harvest and quality traits, our work invites developing novel rootstock breeding schemes targeting fruit quality. It is predictable that in the short run, such efforts will allow the establishment of seed orchards, while improving the gene pool and traceability of seedling rootstocks from “plus tree” donors through nurseries in neotropical regions. In the long term, they will enable identifying *Phytophthora* root rot-resistant, locally adapted, elite rootstock candidates for clonal propagation, as is nowadays routinely performed in temperate regions where avocado trees are not native, and introgression from the wild is controllable.

## Data Availability Statement

The datasets presented in this study can be found in online repositories. The names of the repository/repositories and accession number(s) can be found in the article/Supplementary Material. A preprint of this article is available at the bioRxiv repository (Reyes-Herrera et al., 2020).

## Author Contributions

CD-D, OD-P, and AN-A conceived the original sampling, led phenotypic data collection, and root sampling. VV-Z and LP performed DNA extraction and SSR genotyping and alleles size estimation. PR-H, LM-B, OD-P, and AC filtered and prepared input datasets. AC, LM-B, and PR-H carried out data analyses. AC, OD-P, LM-B, AN-A, and PR-H interpreted results. AC and PR-H drafted a first version of this manuscript, edited by the other co-authors. All authors contributed to the article and approved the submitted version.

## Conflict of Interest

The authors declare that the research was conducted in the absence of any commercial or financial relationships that could be construed as a potential conflict of interest.

## Supplementary Material

Supplementary Figure 1 Morphological first set of traits’ distributions across orchards (first column of figure panels) and agroecological regions (second column of figure panels) for four morphological traits (rows)—tree, trunk, rootstock, and scion heights—recorded in 2016 in “Hass” avocado trees grafted on seedling rootstocks at eight orchards. Orchards spanned three agroecological regions, two in the dairy Northern Andean highland plateau (in green), four in the Eastern Andean highland plateau (in red), and two in the Southwest coffee region (in orange). Abbreviated orchard codes depicted in the *x*-axis of the first column of figure panels are last letters of full names from Figure 1.

Supplementary Figure 2 (Continued from Supplementary Figure S1) Morphological second set of traits’ distributions across orchards (first column of figure panels) and agroecological regions (second column of figure panels) for other four morphological traits (rows)—rootstock and scion perimeters, trunk perimeter at the grafting scar, and rootstock compatibility following (Webber, 1948)—recorded in 2016 in “Hass” avocado trees grafted on seedling rootstocks at eight orchards. Orchards spanned three agroecological regions, two in the dairy Northern Andean highland plateau (in green), four in the Eastern Andean highland plateau (in red), and two in the Southwest coffee region (in orange). Abbreviated orchard codes depicted in the *x*-axis of the first column of figure panels are last letters of full names from Figure 1.

Supplementary Figure 3 Biomass/reproductive traits’ distributions across orchards (first column of figure panels) and agroecological regions (second column of figure panels) for three biomass/reproductive traits (rows)—number of leaves, flowers, and fruits following Salazar-García et al. (2013)—recorded from 2015 to 2016 in “Hass” avocado trees grafted on seedling rootstocks at eight orchards. Orchards spanned three agroecological regions, two in the dairy Northern Andean highland plateau (in green), four in the Eastern Andean highland plateau (in red), and two in the Southwest coffee region (in orange). Abbreviated orchard codes in the first column of figure panels are last letters of full names in Figure 1.

Supplementary Figure 4 Harvest first set of traits’ distributions across orchards (first column of figure panels) and agroecological regions (second column of figure panels) for five harvest traits (rows)—number of fruits with exportation quality and those discarded because of mechanical damage, sun damage, and damage caused by scarab beetles (*A. pygidialis*) or thrips (*F. gardeniae*)—recorded from 2015 to 2016 in “Hass” avocado trees grafted on seedling rootstocks at eight orchards. Orchards spanned three agroecological regions, two in the dairy Northern Andean highland plateau (in green), four in the Eastern Andean highland plateau (in red), and two in the Southwest coffee region (in orange). Abbreviated orchard codes in the first column of figure panels are last letters of full names in Figure 1.

Supplementary Figure 5 (Continued from Supplementary Figure S4) Harvest second set of traits’ distributions across orchards (first column of figure panels) and agroecological regions (second column of figure panels) for other four harvest traits (rows)—number of fruits discarded because damage caused by *Monalonion* spp. or due to other imperfections such as low weight, early ripening or the stalk cut below the pedicel—recorded from 2015 to 2016 in “Hass” avocado trees grafted on seedling rootstocks at eight orchards. Orchards spanned three agroecological regions, two in the dairy Northern Andean highland plateau (in green), four in the Eastern Andean highland plateau (in red), and two in the Southwest coffee region (in orange). Abbreviated orchard codes in the first column of figure panels are last letters of full names in Figure 1.

Supplementary Figure 6 Pearson correlations among eight morphological traits recorded in 2016 in “Hass” avocado trees grafted on seedling rootstocks at eight orchards. Correlation estimates and 95% confidence intervals are presented above the diagonal and below diagonal cells are colored accordingly. Minimum and maximum values are shown in the corners of the cells in the diagonal.

Supplementary Figure 7 Pearson correlations among average distributions of three biomass/reproductive traits recorded from 2015 to 2016 in “Hass” avocado trees grafted on seedling rootstocks at eight orchards. Correlation estimates and 95% confidence intervals are presented above the diagonal and below diagonal cells are colored accordingly. Minimum and maximum values are shown in the corners of the cells in the diagonal.

Supplementary Figure 8 Pearson correlations among average distributions of nine harvest traits recorded from 2015 to 2016 in “Hass” avocado trees grafted on seedling rootstocks at eight orchards. Correlation estimates and 95% confidence intervals are presented above the diagonal and below diagonal cells are colored accordingly. Minimum and maximum values are shown in the corners of the cells in the diagonal.

Supplementary Figure 9 Evanno’s delta *K* for the unsupervised Bayesian genetic clustering conducted in STRUCTURE and depicted in Figure 2. *K* values ranged from *K* = 2 to *K* = 5. Transformed likelihoods of the graph model from the Evanno et al. (2005) are shown in the vertical axis.

Supplementary Figure 10 Posterior distributions for the rootstock-mediated heritability (*h*^2^) estimates per agroecological region. Estimates were gathered using a “genetic prediction” additive mixed linear model according to de los Campos et al. (2009), calibrated with Lynch and Ritland (1999)’s relatedness matrix among rootstocks from eight “Hass” avocado orchards. Depicted traits (rows) are those for which significant *p*-values (*p* < 0.05) were simultaneously obtained for three different permutation strategies (of the phenotypic vector, the matrix of molecular markers and the matrix of genetic relatedness among rootstocks, Table 1). Columns of figure panels respectively show the posterior distribution for the rootstock-mediated heritability (*h*^2^) estimates when computed in the dairy Northern Andean highland plateau (green), the Eastern Andean highland plateau (red), and the Southwest coffee region (orange). Dashed vertical gray lines and continuous horizontal gray lines respectively mark mean values and 95% confidence intervals. Lines are colored according to Figure 1.

Supplementary Figure 11 Estimates of narrow-sense rootstock-mediated heritability (*h*^2^) in five of the 20 measured traits based on a “genetic prediction” model calibrated with 13 SSRs markers genotyped in seedling rootstocks from eight “Hass” avocado orchards. Depicted traits (rows) are those for which significant *p*-values (*p* < 0.05) were simultaneously obtained for three different permutation strategies (of the phenotypic vector, the matrix of molecular markers and the matrix of genetic relatedness among rootstocks, Table 1). The first column of figure panels shows the posterior distribution for the rootstock-mediated heritability (*h*^2^) estimates as well as their mean (dashed vertical gray line) and 95% confidence interval (continuous horizontal gray line). The second column of figure panels reflects the model fits (*r*) expressed as the correlation between the observed trait phenotype (*y*_*i*_) and the model’s trait estimation (β*x*_*i*_)—(Eq. 3). The third column of figure panels recalls the trait distribution across orchards (from Figure 4 and Supplementary Figures S1–S5). Estimates of *h*^2^ and *r* are derived from an additive mixed linear model according to de los Campos et al. (2009).

Supplementary Table 1 Phenotypic and genetic data of 240 “Hass” avocado trees grafted on seedling rootstocks from eight orchards in the northwest of Colombia (province of Antioquia). Orchards were distributed across three agroecological regions, two in the dairy Northern Andean highland plateau, four in the Eastern Andean highland plateau, and two in the Southwest coffee region. From each orchard, 30 healthy trees from six linear blocks were chosen. Eight morphological traits were recorded in 2016, while three biomass/reproductive and nine harvest traits were measured from 2015 to 2017. For these last 12 traits average values across all 3 years are shown. Rootstocks were genotyped for 13 SSR markers (Supplementary Table S2) from Sharon et al. (1997) and Ashworth et al. (2004). Alleles sizes are kept.

Supplementary Table 2 Identity of the 13 microsatellite markers (simple sequence repeats—SSRs) used in this study to screen rootstocks from eight “Hass” avocado orchards. Forward and reverse primers, sequence motif, source and summary statistics are shown. Markers were originally designed by Sharon et al. (1997) and Ashworth et al. (2004), and were prioritized according to their polymorphism information content (PIC), following Alcaraz and Hormaza (2007).

Supplementary Table 3 Wilcoxon Rank Sum Test results comparing trait distributions across agroecological regions for the 20 traits surveyed at eight orchards of “Hass” avocado trees grafted on seedling rootstocks. Orchards spanned three agroecological regions, two in the dairy Northern Andean highland plateau (heading in green), four in the Eastern Andean highland plateau (heading in red), and two in the Southwest coffee region (heading in orange). Traits in bold had significant heritability (*h*^2^) estimates (*p* < 0.05) for three different permutation strategies (of the phenotypic vector, the matrix of molecular markers and the matrix of genetic relatedness among rootstocks, Table 1).

Supplementary Table 4 Wilcoxon Rank Sum Test results comparing trait distributions across eight orchards of “Hass” avocado and examined for 20 traits within regions. Orchards spanned three agroecological regions, two in the dairy Northern Andean highland plateau (heading in green), four in the Eastern Andean highland plateau (heading in red), and two in the Southwest coffee region (heading in orange). Traits in bold had significant heritability (*h*^2^) estimates (*p* < 0.05) for three different permutation strategies (of the phenotypic vector, the matrix of molecular markers and the matrix of genetic relatedness among rootstocks, Table 1).

Supplementary Table 5 Pearson correlation coefficients comparing trait distributions of 20 traits examined across eight orchards of “Hass” avocado and altitude. Orchards differed in altitude (Figure 1) and spanned three agroecological regions, two in the dairy Northern Andean highland plateau (heading in green), four in the Eastern Andean highland plateau (heading in red), and two in the Southwest coffee region (heading in orange). Traits in bold had significant heritability (*h*^2^) estimates (*p* < 0.05) for three different permutation strategies (of the phenotypic vector, the matrix of molecular markers and the matrix of genetic relatedness among rootstocks, Table 1).

Supplementary Table 6 *Q*-matrix and admixture index in rootstocks from eight orchards of “Hass” avocado as determined by 13 SSR markers. Estimates are derived from the STRUCTURE software (Pritchard et al., 2000) at *K* = 3 (Figure 2).
