# Supplementary figures and images for: Inheritance of Rootstock Effects in Avocado (Persea americana Mill.) cv. Hass
Source: Front Plant Sci. 2020 Dec 23;11:555071. doi: 10.3389/fpls.2020.555071 (PMC7785968; doi:10.3389/fpls.2020.555071)

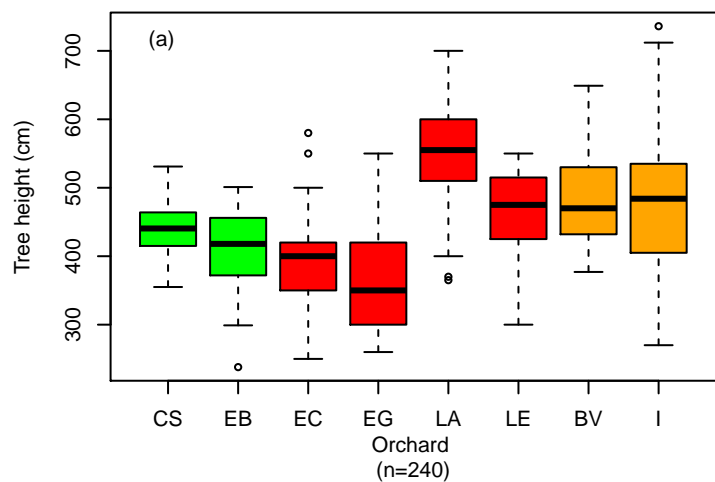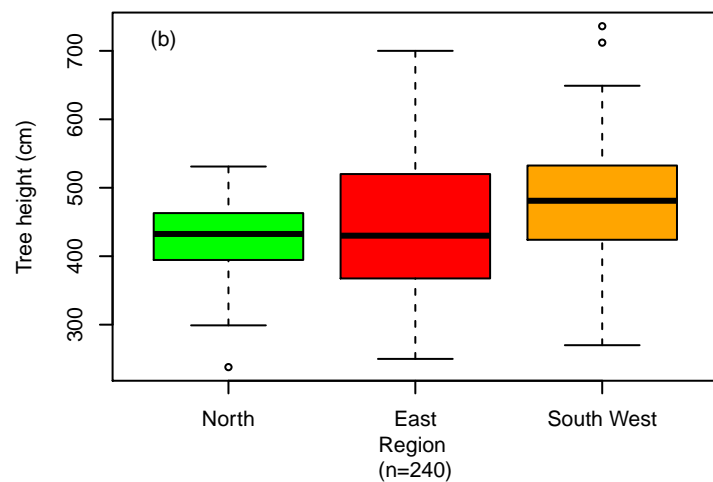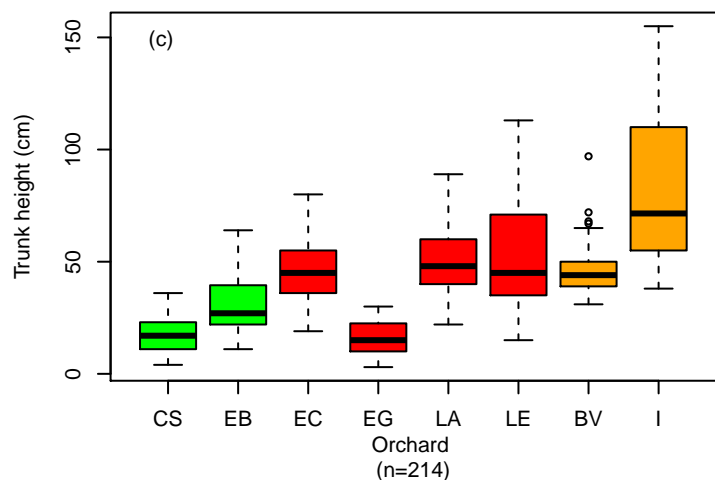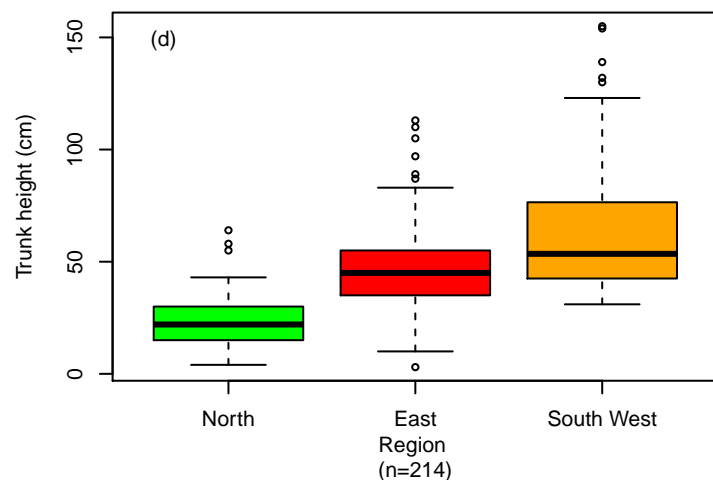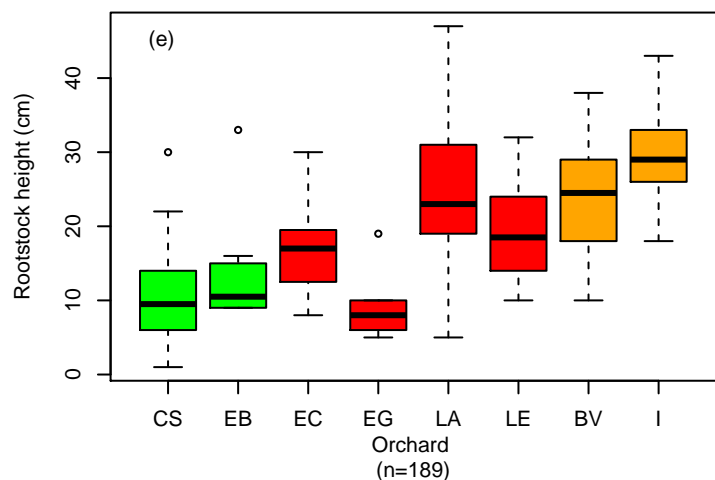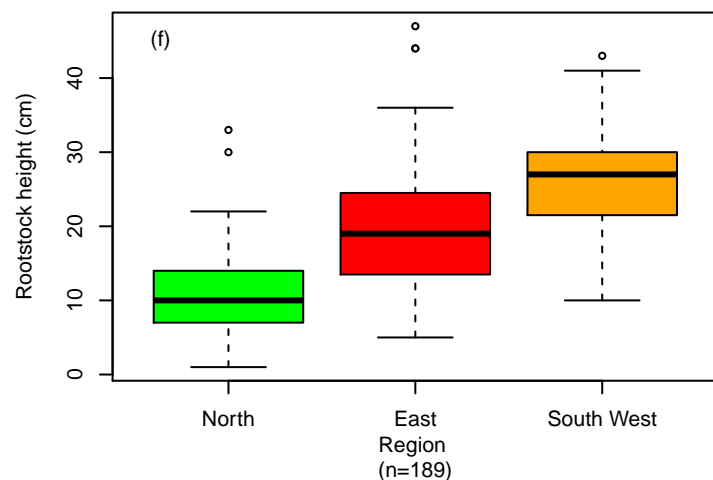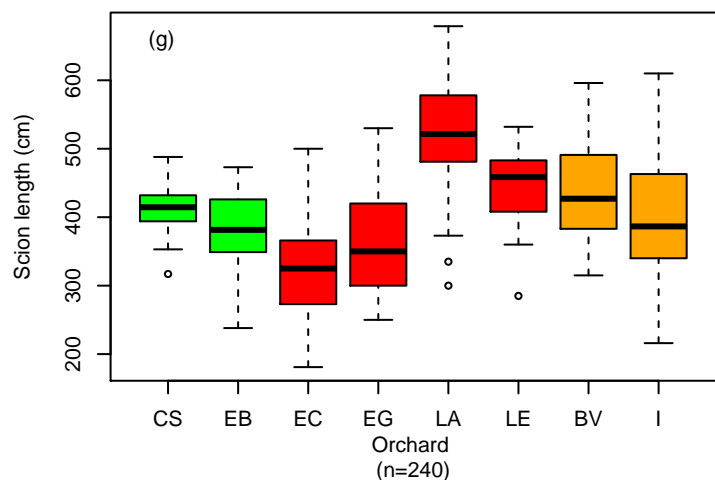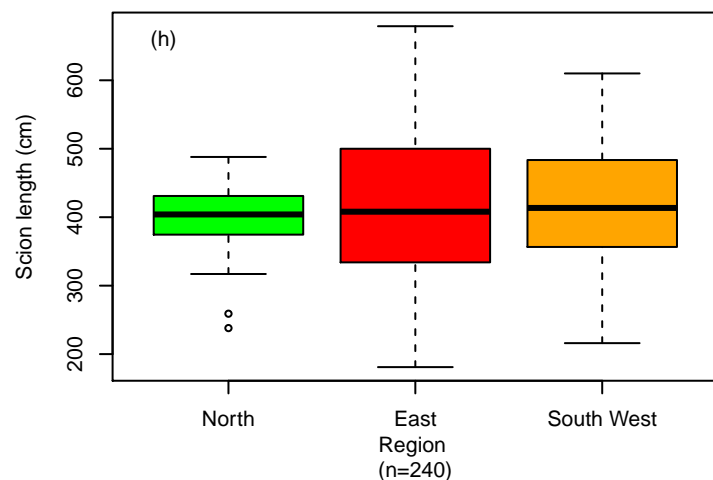

Supplement: Supplementary Figure 1 — Morphological first set of traits’ distributions across orchards (first column of figure panels) and agroecological regions (second column of figure panels) for four morphological traits (rows)—tree, trunk, rootstock, and scion heights—recorded in 2016 in “Hass” avocado trees grafted on seedling rootstocks at eight orchards. Orchards spanned three agroecological regions, two in the dairy Northern Andean highland plateau (in green), four in the Eastern Andean highland plateau (in red), and two in the Southwest coffee region (in orange). Abbreviated orchard codes depicted in the x-axis of the first column of figure panels are last letters of full names from Figure 1. [file Data_Sheet_1.PDF]

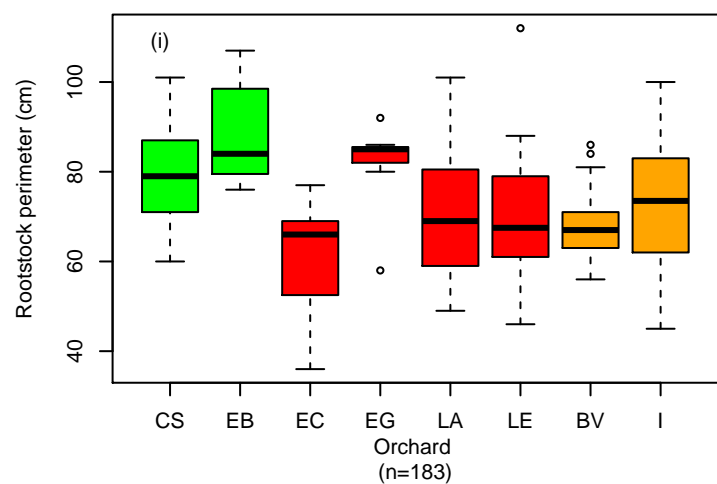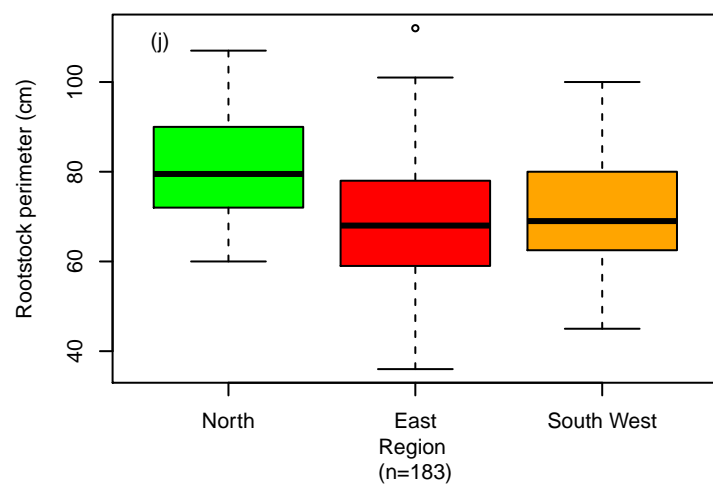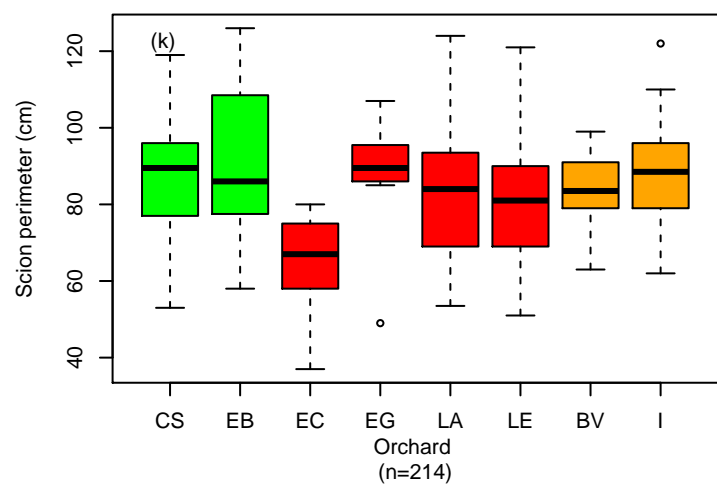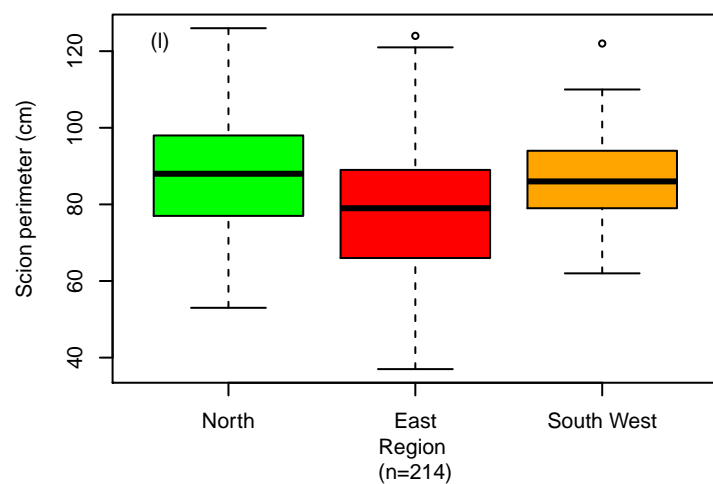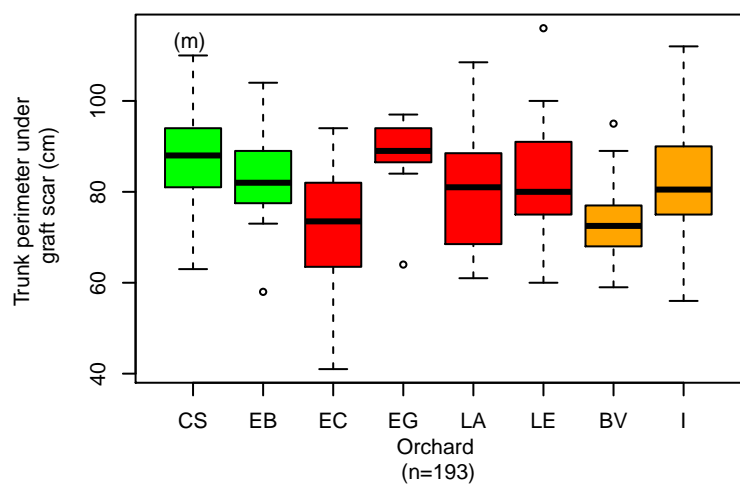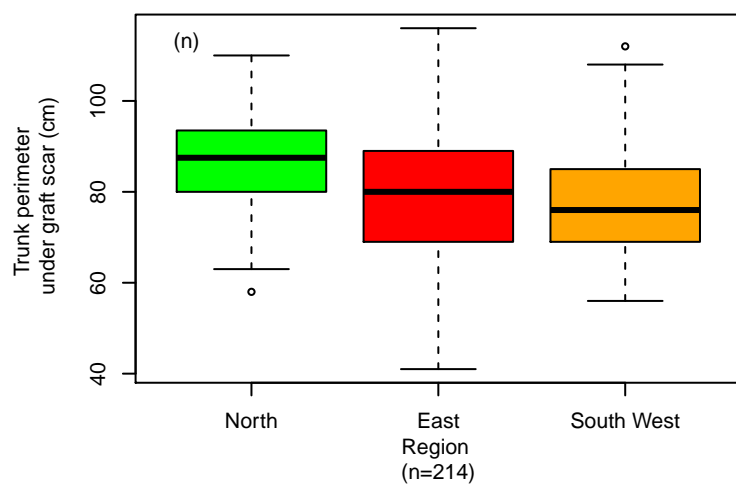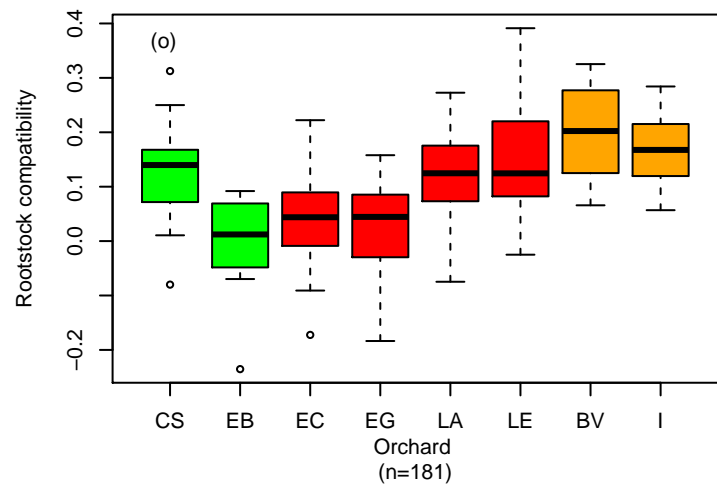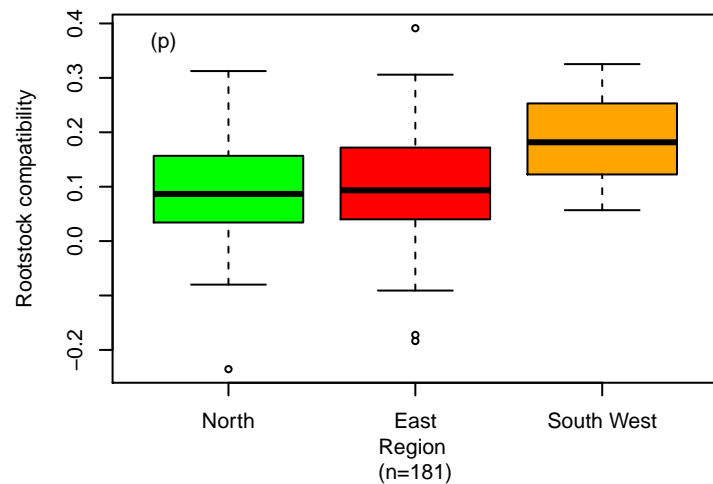

Supplement: Supplementary Figure 2 — (Continued from Supplementary Figure S1) Morphological second set of traits’ distributions across orchards (first column of figure panels) and agroecological regions (second column of figure panels) for other four morphological traits (rows)—rootstock and scion perimeters, trunk perimeter at the grafting scar, and rootstock compatibility following (Webber, 1948)—recorded in 2016 in “Hass” avocado trees grafted on seedling rootstocks at eight orchards. Orchards spanned three agroecological regions, two in the dairy Northern Andean highland plateau (in green), four in the Eastern Andean highland plateau (in red), and two in the Southwest coffee region (in orange). Abbreviated orchard codes depicted in the x-axis of the first column of figure panels are last letters of full names from Figure 1. [file Data_Sheet_2.PDF]

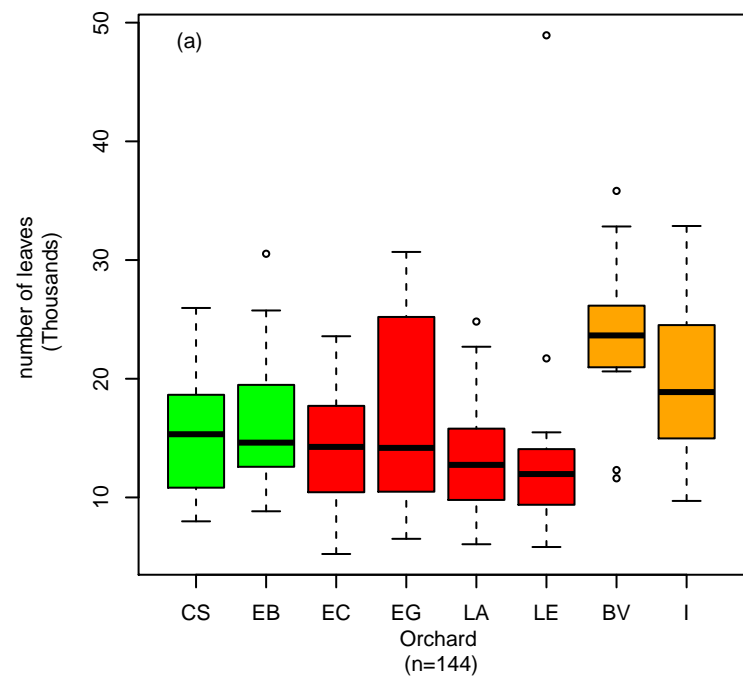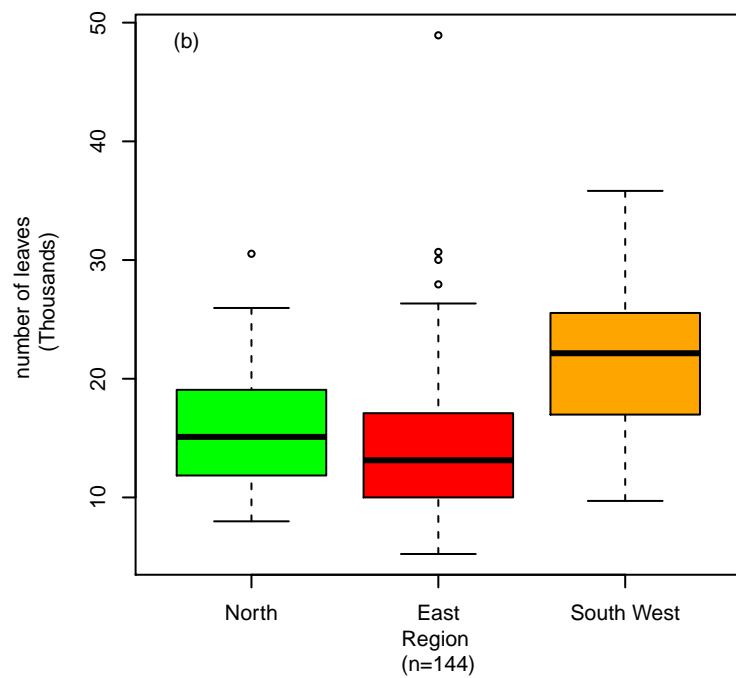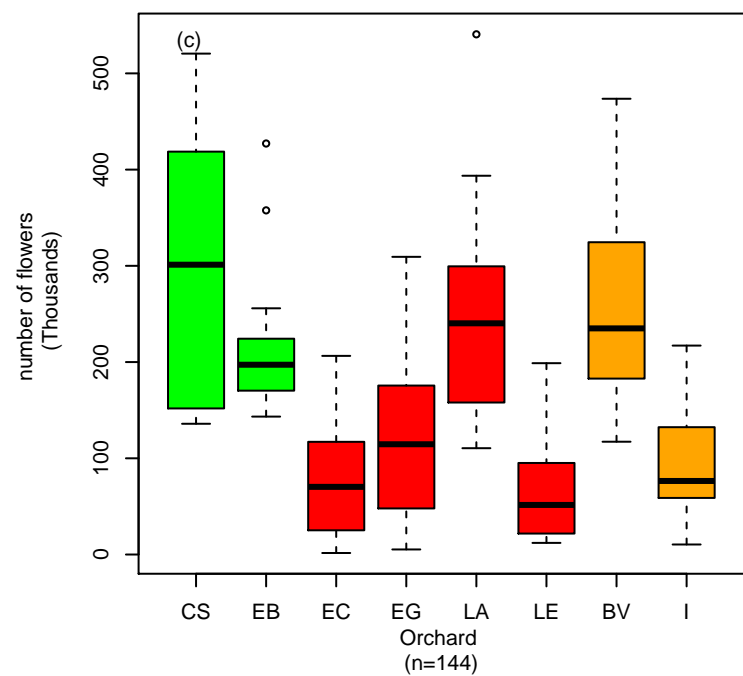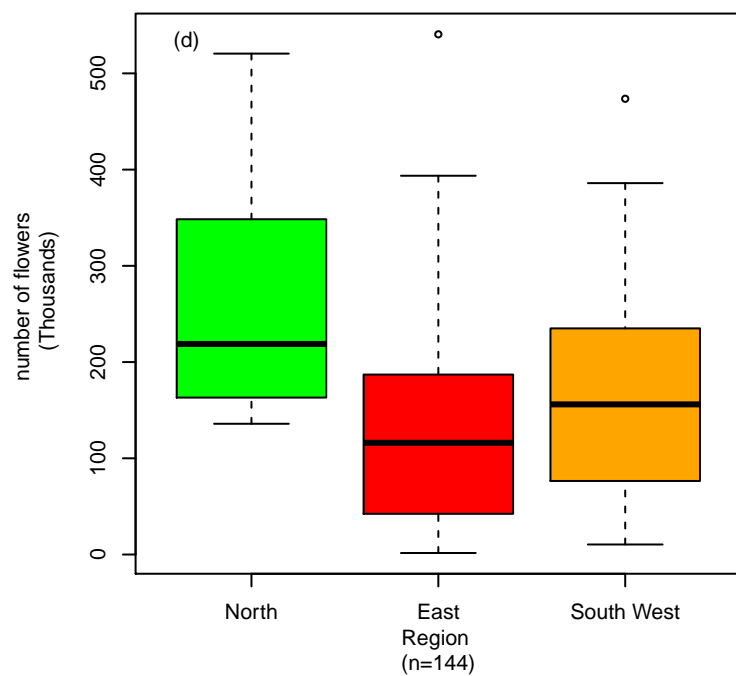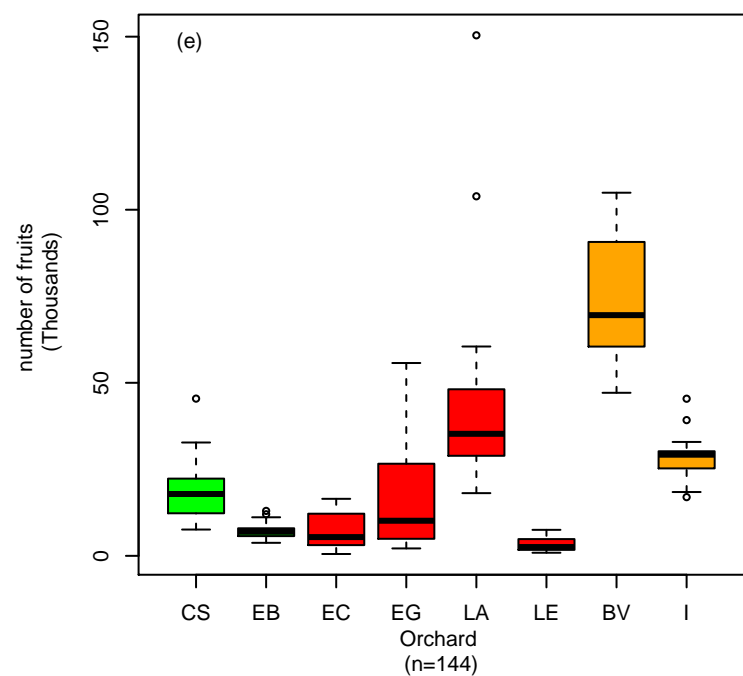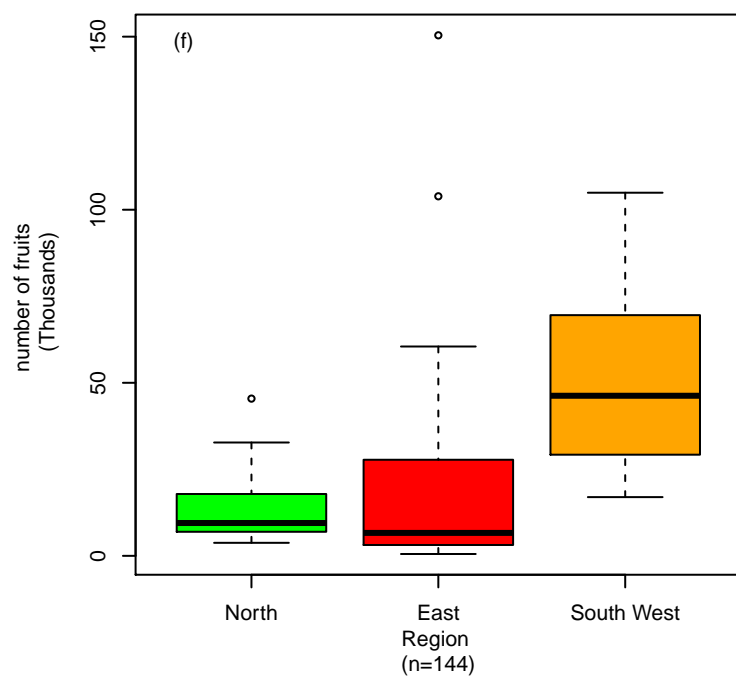

Supplement: Supplementary Figure 3 — Biomass/reproductive traits’ distributions across orchards (first column of figure panels) and agroecological regions (second column of figure panels) for three biomass/reproductive traits (rows)—number of leaves, flowers, and fruits following Salazar-García et al. (2013)—recorded from 2015 to 2016 in “Hass” avocado trees grafted on seedling rootstocks at eight orchards. Orchards spanned three agroecological regions, two in the dairy Northern Andean highland plateau (in green), four in the Eastern Andean highland plateau (in red), and two in the Southwest coffee region (in orange). Abbreviated orchard codes in the first column of figure panels are last letters of full names in Figure 1. [file Data_Sheet_3.PDF]

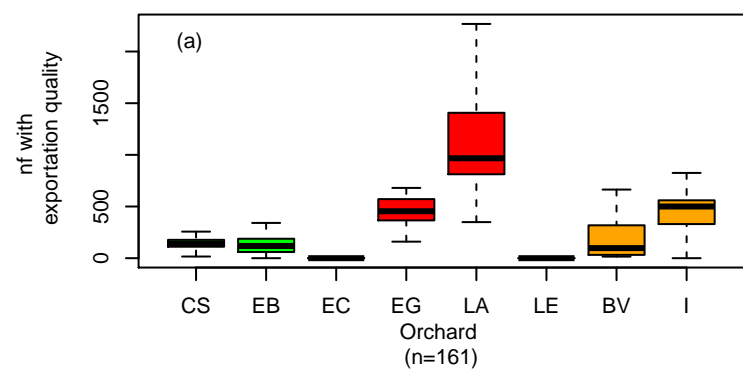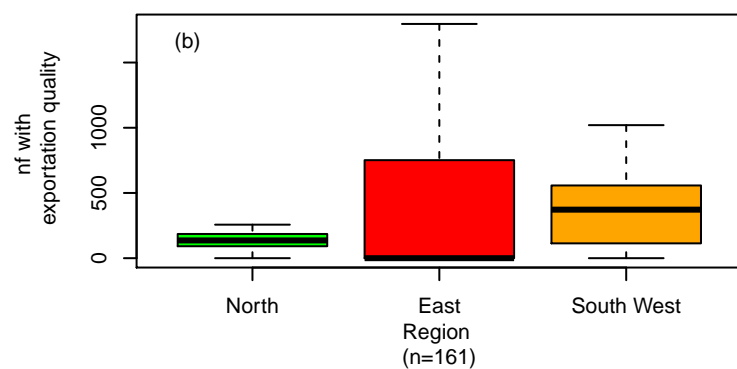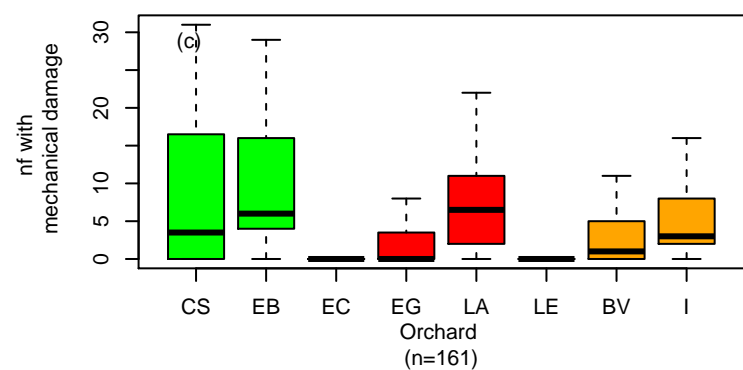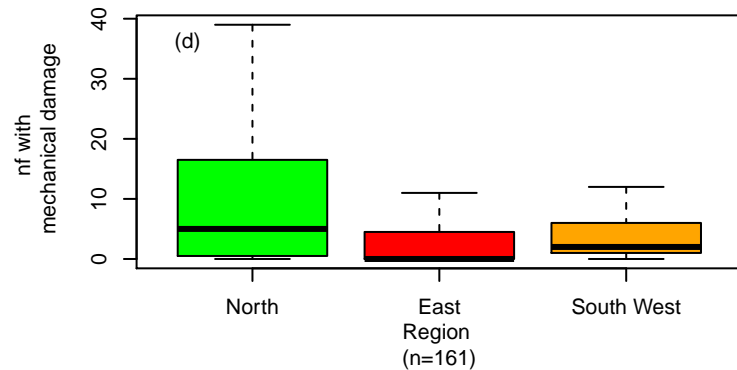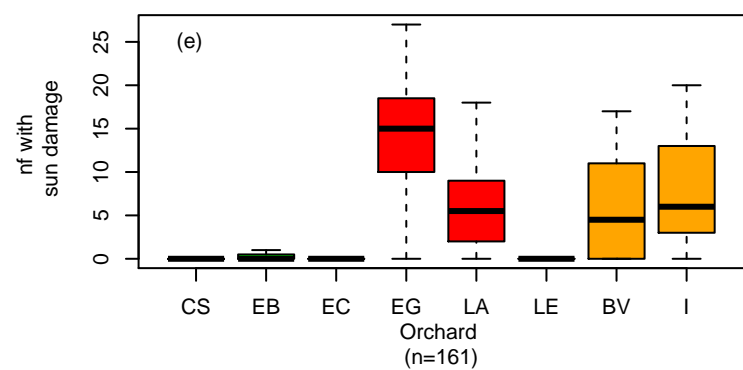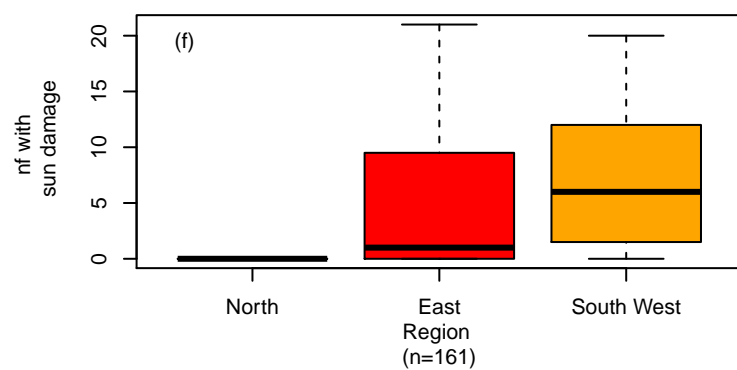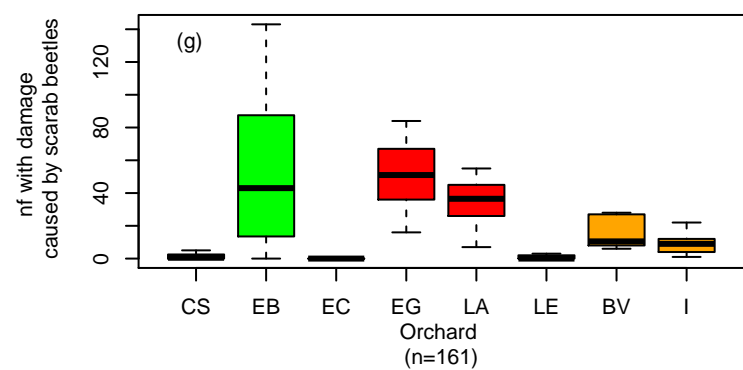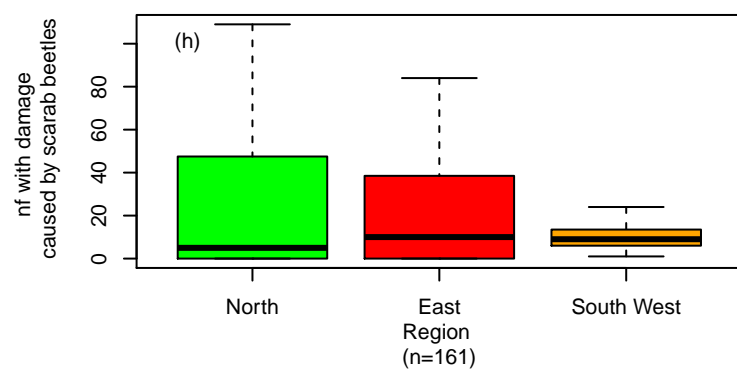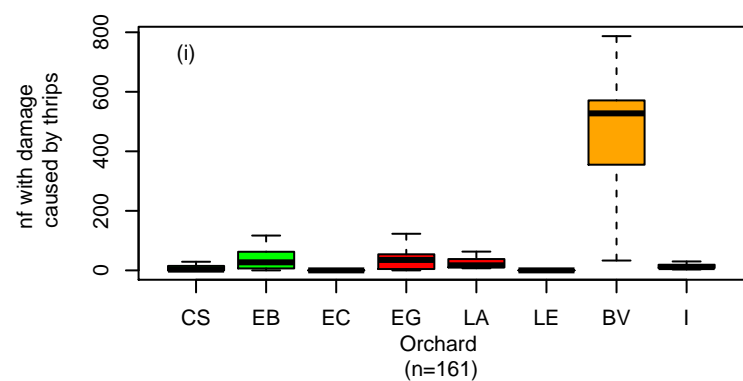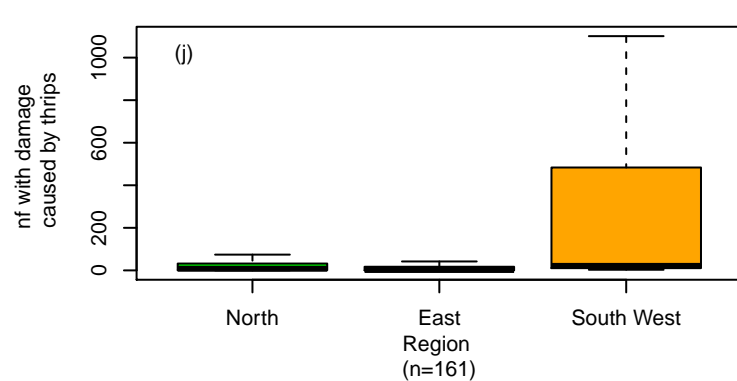

Supplement: Supplementary Figure 4 — Harvest first set of traits’ distributions across orchards (first column of figure panels) and agroecological regions (second column of figure panels) for five harvest traits (rows)—number of fruits with exportation quality and those discarded because of mechanical damage, sun damage, and damage caused by scarab beetles (A. pygidialis) or thrips (F. gardeniae)—recorded from 2015 to 2016 in “Hass” avocado trees grafted on seedling rootstocks at eight orchards. Orchards spanned three agroecological regions, two in the dairy Northern Andean highland plateau (in green), four in the Eastern Andean highland plateau (in red), and two in the Southwest coffee region (in orange). Abbreviated orchard codes in the first column of figure panels are last letters of full names in Figure 1. [file Data_Sheet_4.PDF]

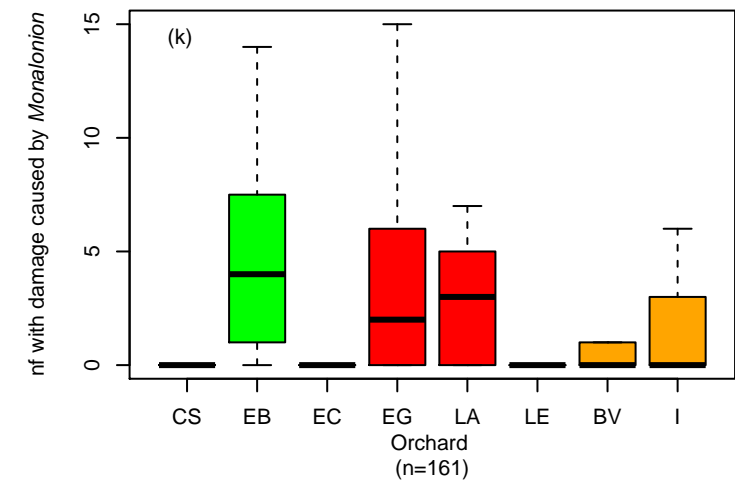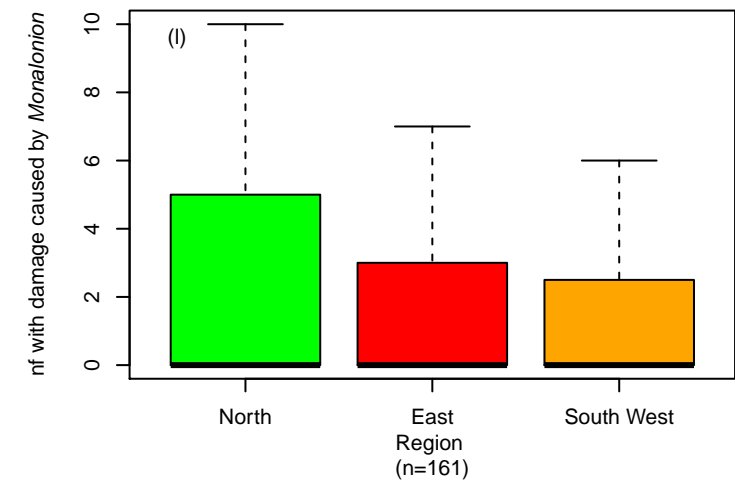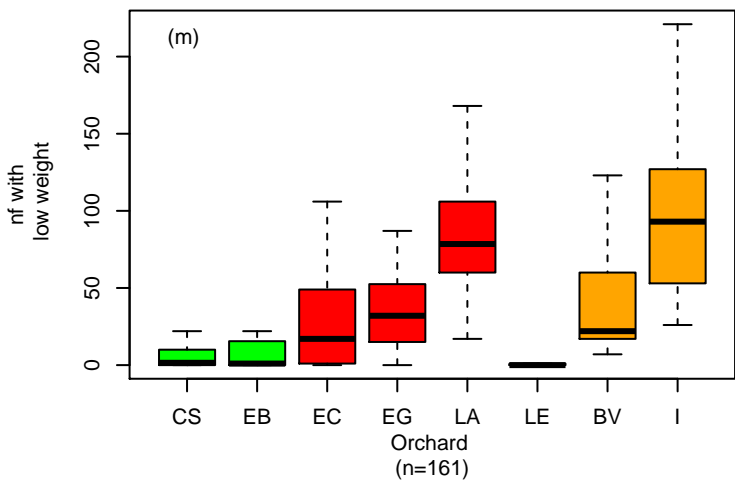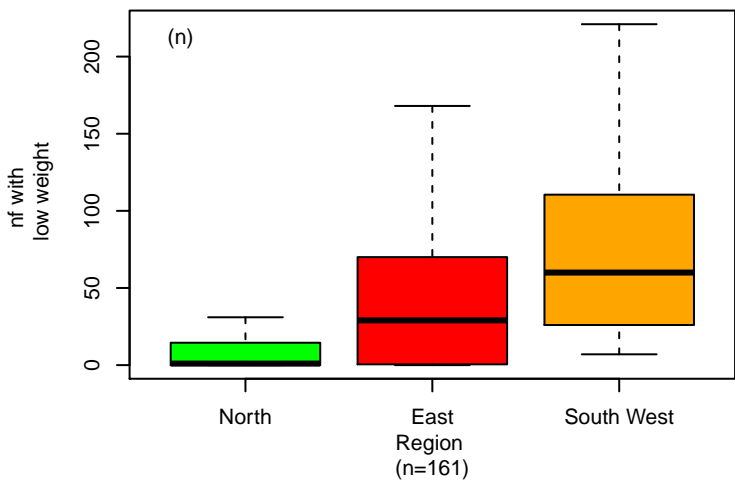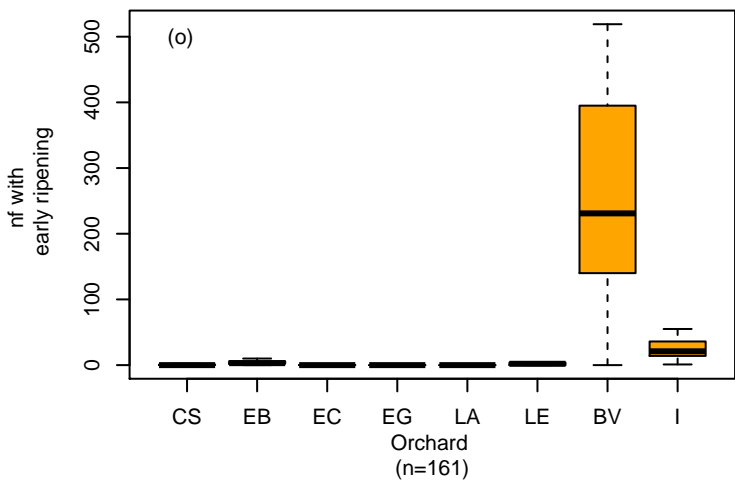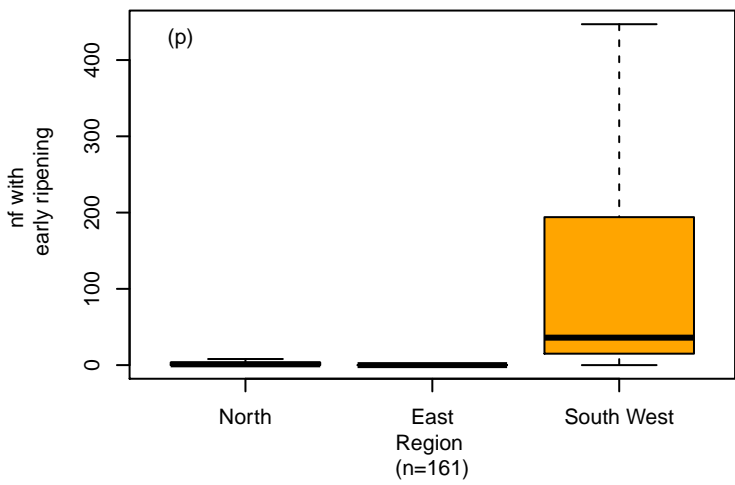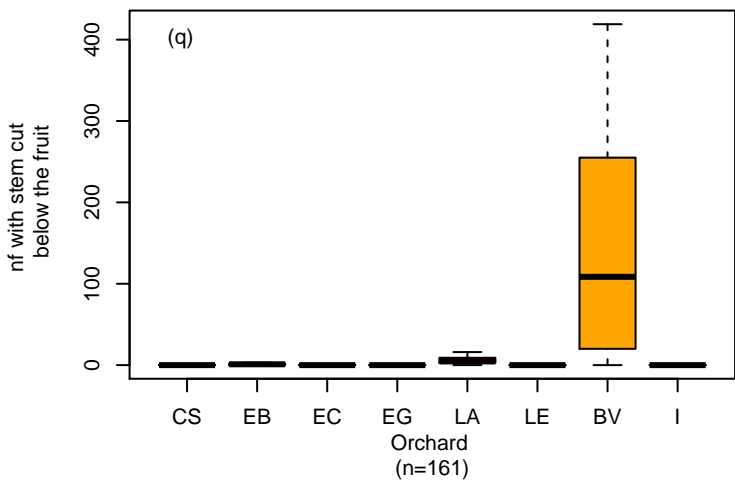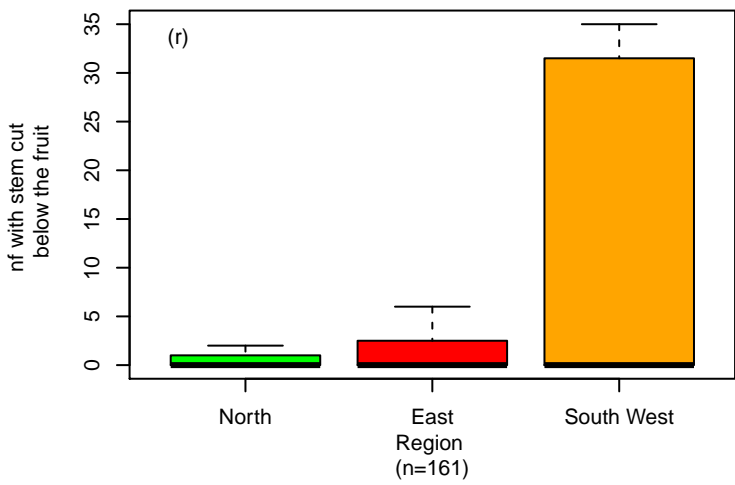

Supplement: Supplementary Figure 5 — (Continued from Supplementary Figure S4) Harvest second set of traits’ distributions across orchards (first column of figure panels) and agroecological regions (second column of figure panels) for other four harvest traits (rows)—number of fruits discarded because damage caused by Monalonion spp. or due to other imperfections such as low weight, early ripening or the stalk cut below the pedicel—recorded from 2015 to 2016 in “Hass” avocado trees grafted on seedling rootstocks at eight orchards. Orchards spanned three agroecological regions, two in the dairy Northern Andean highland plateau (in green), four in the Eastern Andean highland plateau (in red), and two in the Southwest coffee region (in orange). Abbreviated orchard codes in the first column of figure panels are last letters of full names in Figure 1. [file Data_Sheet_5.PDF]

Morphological Traits

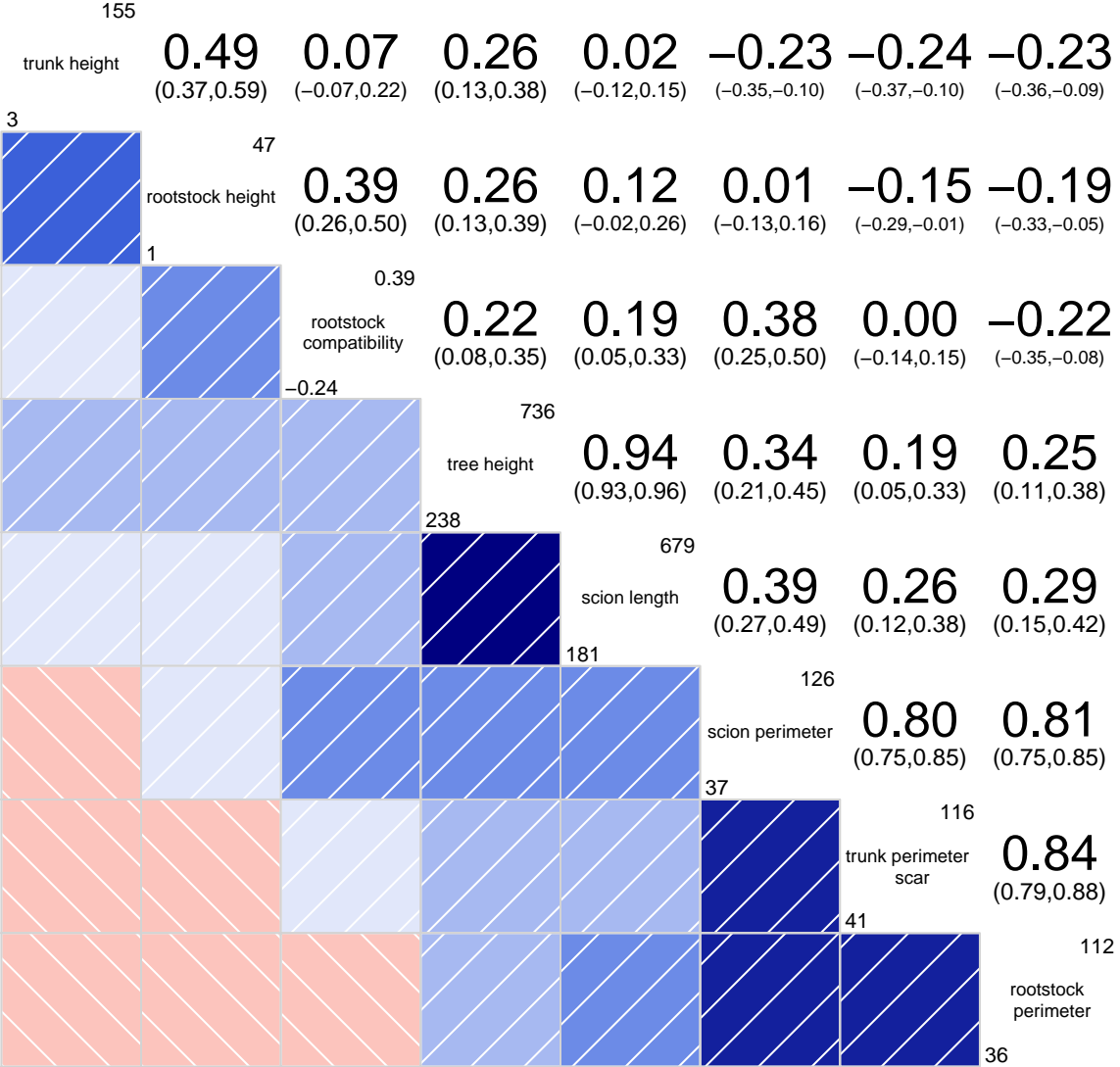

Supplement: Supplementary Figure 6 — Pearson correlations among eight morphological traits recorded in 2016 in “Hass” avocado trees grafted on seedling rootstocks at eight orchards. Correlation estimates and 95% confidence intervals are presented above the diagonal and below diagonal cells are colored accordingly. Minimum and maximum values are shown in the corners of the cells in the diagonal. [file Data_Sheet_6.PDF]

Ecophysiological Traits

150394

number of fruits

0.40  
(0.25,0.53)

0.37  
(0.22,0.51)

535

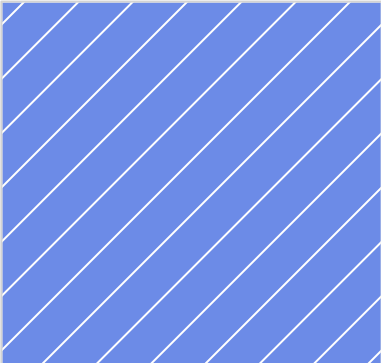

48933.04

number of leaves

0.38  
(0.24,0.52)

5236.2

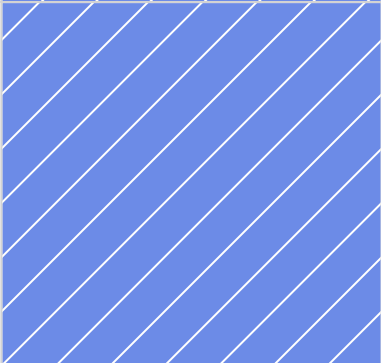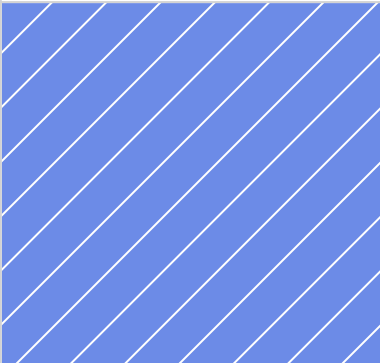

540596

number of flowers

1584

Supplement: Supplementary Figure 7 — Pearson correlations among average distributions of three biomass/reproductive traits recorded from 2015 to 2016 in “Hass” avocado trees grafted on seedling rootstocks at eight orchards. Correlation estimates and 95% confidence intervals are presented above the diagonal and below diagonal cells are colored accordingly. Minimum and maximum values are shown in the corners of the cells in the diagonal. [file Data_Sheet_7.PDF]

## Harvest Traits

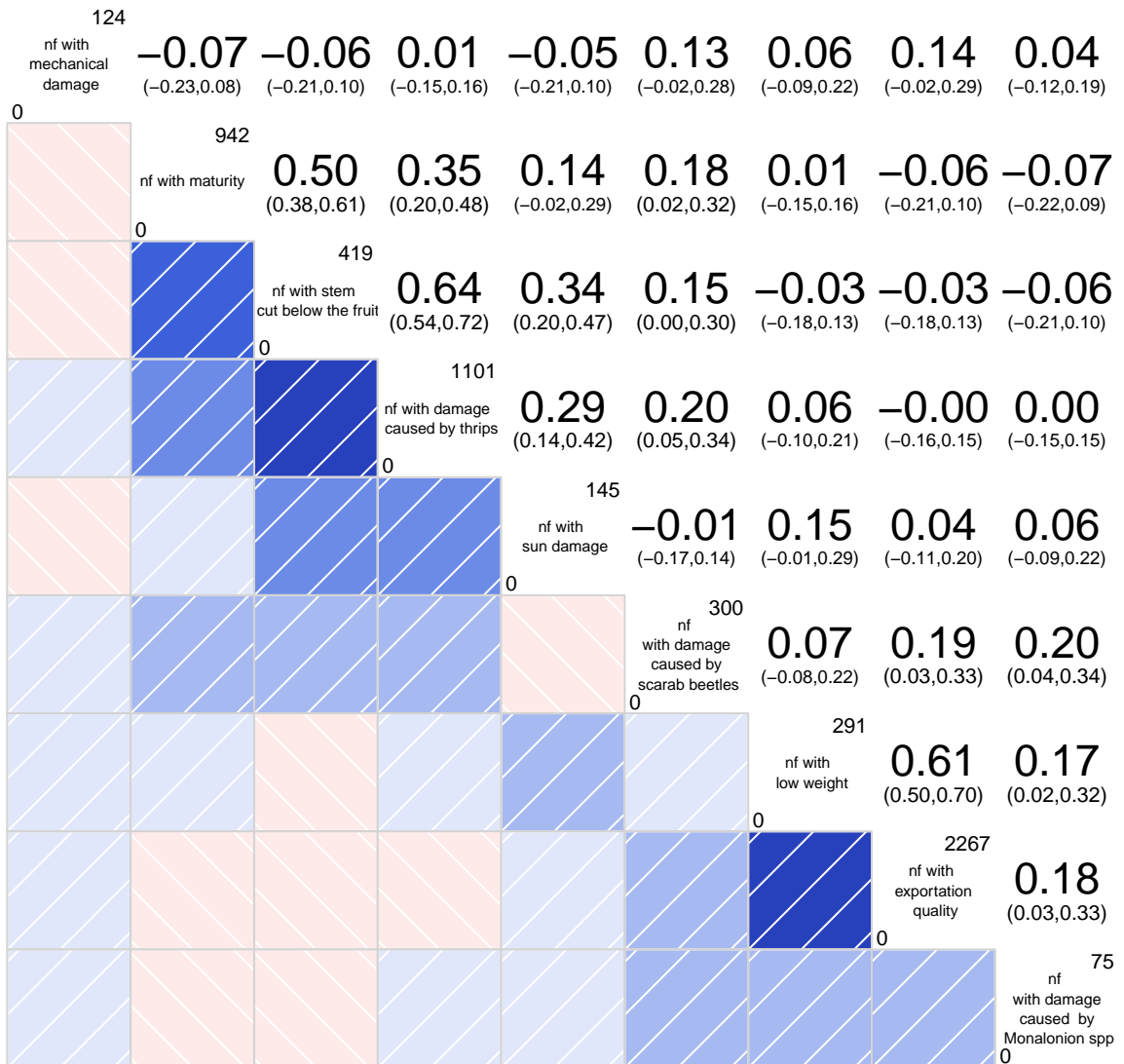

Supplement: Supplementary Figure 8 — Pearson correlations among average distributions of nine harvest traits recorded from 2015 to 2016 in “Hass” avocado trees grafted on seedling rootstocks at eight orchards. Correlation estimates and 95% confidence intervals are presented above the diagonal and below diagonal cells are colored accordingly. Minimum and maximum values are shown in the corners of the cells in the diagonal. [file Data_Sheet_8.PDF]

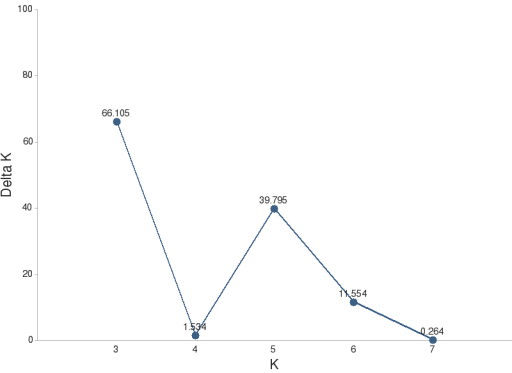

Supplement: Supplementary Figure 9 — Evanno’s delta K for the unsupervised Bayesian genetic clustering conducted in STRUCTURE and depicted in Figure 2. K values ranged from K = 2 to K = 5. Transformed likelihoods of the graph model from the Evanno et al. (2005) are shown in the vertical axis. [file Data_Sheet_9.PDF]

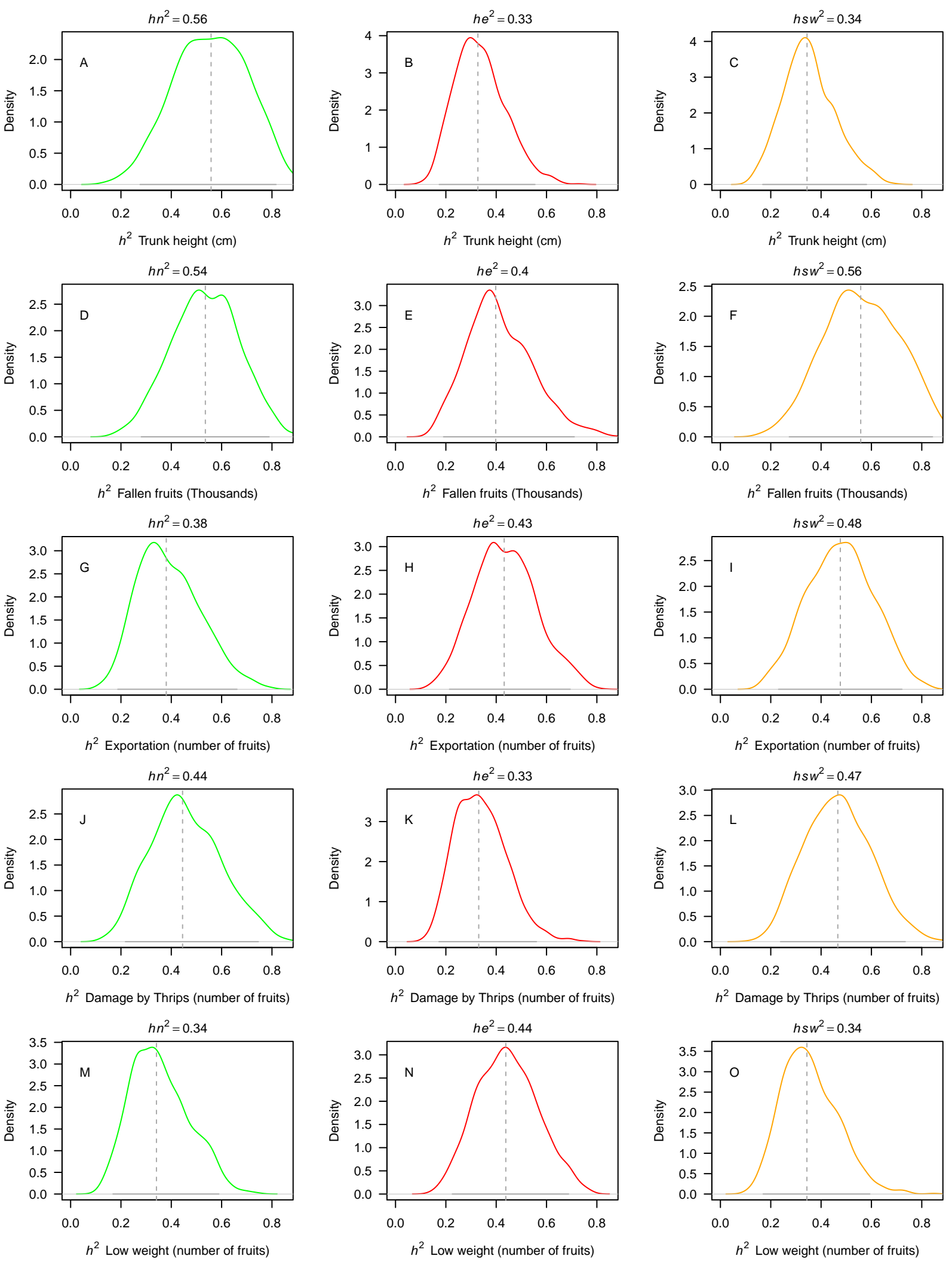

Supplement: Supplementary Figure 10 — Posterior distributions for the rootstock-mediated heritability (h2) estimates per agroecological region. Estimates were gathered using a “genetic prediction” additive mixed linear model according to de los Campos et al. (2009), calibrated with Lynch and Ritland (1999)’s relatedness matrix among rootstocks from eight “Hass” avocado orchards. Depicted traits (rows) are those for which significant p-values (p < 0.05) were simultaneously obtained for three different permutation strategies (of the phenotypic vector, the matrix of molecular markers and the matrix of genetic relatedness among rootstocks, Table 1). Columns of figure panels respectively show the posterior distribution for the rootstock-mediated heritability (h2) estimates when computed in the dairy Northern Andean highland plateau (green), the Eastern Andean highland plateau (red), and the Southwest coffee region (orange). Dashed vertical gray lines and continuous horizontal gray lines respectively mark mean values and 95% confidence intervals. Lines are colored according to Figure 1. [file Data_Sheet_10.PDF]

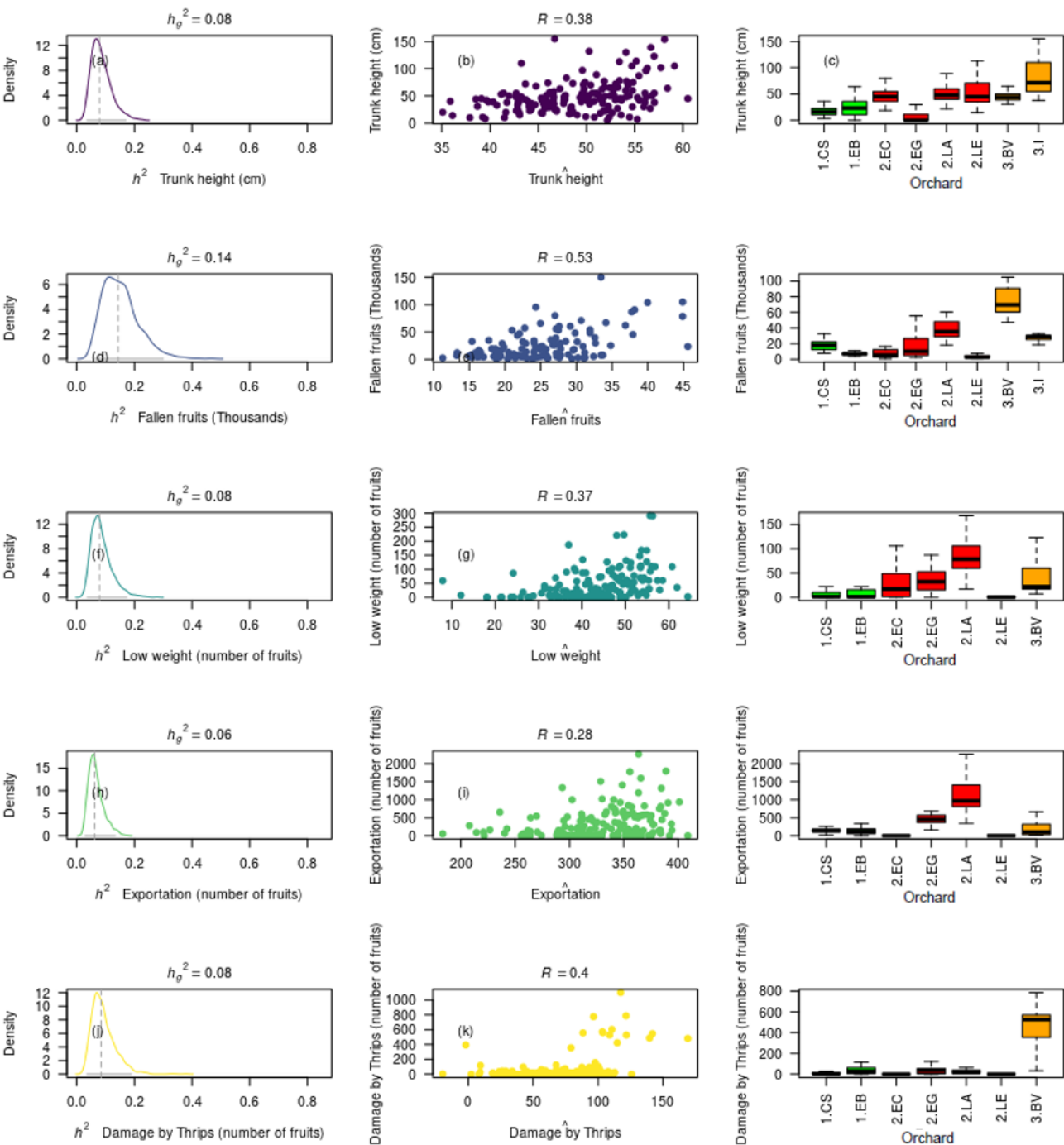

Supplement: Supplementary Figure 11 — Estimates of narrow-sense rootstock-mediated heritability (h2) in five of the 20 measured traits based on a “genetic prediction” model calibrated with 13 SSRs markers genotyped in seedling rootstocks from eight “Hass” avocado orchards. Depicted traits (rows) are those for which significant p-values (p < 0.05) were simultaneously obtained for three different permutation strategies (of the phenotypic vector, the matrix of molecular markers and the matrix of genetic relatedness among rootstocks, Table 1). The first column of figure panels shows the posterior distribution for the rootstock-mediated heritability (h2) estimates as well as their mean (dashed vertical gray line) and 95% confidence interval (continuous horizontal gray line). The second column of figure panels reflects the model fits (r) expressed as the correlation between the observed trait phenotype (yi) and the model’s trait estimation (βxi)—(Eq. 3). The third column of figure panels recalls the trait distribution across orchards (from Figure 4 and Supplementary Figures S1–S5). Estimates of h2 and r are derived from an additive mixed linear model according to de los Campos et al. (2009). [file Data_Sheet_11.PDF]
